# The segmented flavivirus Alongshan virus reduces mitochondrial mass by degrading STAT2 to suppress the innate immune response

**DOI:** 10.1128/jvi.01301-24

**Published:** 2024-12-10

**Authors:** Yinghua Zhao, Liyan Sui, Mingming Pan, Fangyu Jin, Yuan Huang, Shu Fang, Mengmeng Wang, Lihe Che, Wenbo Xu, Nan Liu, Haicheng Gao, Zhijun Hou, Fang Du, Zhengkai Wei, Lesley Bell-Sakyi, Jixue Zhao, Kaiyu Zhang, Yicheng Zhao, Quan Liu

**Affiliations:** 1 Department of Infectious Diseases and Center of Infectious Diseases and Pathogen Biology, Key Laboratory of Organ Regeneration and Transplantation of the Ministry of Education, State Key Laboratory for Diagnosis and Treatment of Severe Zoonotic Infectious Diseases, Key Laboratory for Zoonosis of the Ministry of Education, The First Hospital of Jilin University 117971 , Changchun, China; 2 College of Wildlife and Protected Area, Northeast Forestry University 673337 , Harbin, China; 3 School of Pharmaceutical Sciences, Jilin University 623661 , Changchun, China; 4 Department of Neurology, Xijing Hospital, Air Force Medical University 12644 , Xi'an, Shaanxi, China; 5 College of Veterinary Medicine, Southwest University 26463 , Chongqing, China; 6 Department of Infection Biology and Microbiomes, Institute of Infection, Veterinary and Ecological Sciences, University of Liverpool 568406 , Liverpool, United Kingdom; 7 Department of Pediatric Surgery, The First Hospital of Jilin University 629131 , Changchun, China; University of North Carolina at Chapel Hill, Chapel Hill, North Carolina, USA

**Keywords:** segmented flavivirus, Alongshan virus, NSP1, STAT2, mitochondrial mass, innate immune response

## Abstract

Alongshan virus (ALSV) is a newly discovered pathogen in the *Flaviviridae* family, characterized by a unique multi-segmented genome that is distantly related to the canonical flaviviruses. Understanding the pathogenic mechanism of this emerging segmented flavivirus is crucial for the development of effective intervention strategies. In this study, we demonstrate that ALSV can infect various mammalian cells and induce the expression of antiviral genes. Furthermore, ALSV is sensitive to IFN-β, but it has developed strategies to counteract the host’s type I IFN response. Mechanistically, ALSV’s nonstructural protein NSP1 interacts with and degrades human STAT2 through an autophagy pathway, with species-dependent effects. This degradation directly inhibits the expression of interferon-stimulated genes (ISGs). Additionally, NSP1-mediated degradation of STAT2 disrupts mitochondrial dynamics, leading to mitophagy and inhibition of mitochondrial biogenesis. This, in turn, suppresses the host’s innate immune response. Interestingly, we found that inhibiting mitophagy using 3-methyladenine and enhancing mitochondrial biogenesis with the PPARγ agonist pioglitazone can reverse NSP1-mediated inhibition of ISGs, suggesting that promoting mitochondrial mass could serve as an effective antiviral strategy. Specifically, the NSP1 methyltransferase domain binds to the key sites of F175/R176 located in the coiled-coil domain of STAT2. Our findings provide valuable insights into the intricate regulatory cross talk between ALSV and the host’s innate immune response, shedding light on the pathogenesis of this emerging segmented flavivirus and offering potential intervention strategies.

**IMPORTANCE:**

Alongshan virus (ALSV), a segmented flavivirus belonging to the *Flaviviridae* family, was first identified in individuals who had been bitten by ticks in Northeastern China. ALSV infection is responsible for causing Alongshan fever, a condition characterized by various clinical symptoms, including fever, headache, skin rash, myalgia, arthralgia, depression, and coma. There is an urgent need for effective antiviral therapies. Here, we demonstrate that ALSV is susceptible to IFN-β but has developed mechanisms to counteract the host’s innate immune response. Specifically, the ALSV nonstructural protein NSP1 interacts with STAT2, leading to its degradation via an autophagy pathway that exhibits species-dependent effects. Additionally, NSP1 disrupts mitochondrial dynamics and suppresses mitochondrial biogenesis, resulting in a reduction in mitochondrial mass, which ultimately contributes to the inhibition of the host’s innate immune response. Interestingly, we found that inhibiting mitophagy and promoting mitochondrial biogenesis can reverse NSP1-mediated suppression of innate immune response by increasing mitochondrial mass. These findings provide valuable insights into the molecular mechanisms of ALSV pathogenesis and suggest potential therapeutic targets against ALSV infection.

## INTRODUCTION

In recent decades, a diverse range of vector-borne viruses capable of infecting humans and causing diseases has come to light (1, 2). Alongshan virus (ALSV), a segmented flavivirus in the *Flaviviridae* family, was identified in patients who had experienced tick bites in Northeastern China (3, 4). Subsequent studies have revealed the widespread presence of ALSV in various regions, including Russia, Finland, Switzerland, and Germany (5–9). ALSV infection leads to a condition known as Alongshan fever, characterized by common clinical symptoms such as fever, headache, skin rash, myalgia, arthralgia, depression, and coma (3, 4, 10). Importantly, Jingmen tick virus (JMTV), another segmented flavivirus, also demonstrated the ability to infect humans, underscoring the emerging threat posed by segmented flaviviruses to human health (11–13).

Several members of the *Flavivirus* genus are significant for human health, including dengue virus (DENV), West Nile virus (WNV), Zika virus (ZIKV), and yellow fever virus (YFV), transmitted by mosquitoes; and tick-borne encephalitis virus (TBEV), which is a tick-borne flavivirus. These viruses share common characteristics as they are enveloped and carry a positive-sense, single-stranded RNA genome, which consists of a single open reading frame (ORF) that encodes three structural proteins (capsid, membrane, and envelope) and seven nonstructural (NS) proteins (NS1, NS2A, NS2B, NS3, NS4A, NS4B, and NS5) (14–16). However, ALSV and JMTV represent distinct members of the family *Flaviviridae*, as their genome comprises four single-stranded, positive-sense RNA segments. Among these, segments 2 and 4 encode structural proteins VP1-VP4, the evolutionary origin of which remains unknown. Conversely, segments 1 and 3 encode the nonstructural proteins NSP1 and NSP2, which individually exhibit homology to the NS5 and NS2B-NS3 of the mono-segmented flaviviruses (4, 17, 18). Notably, ALSV NSP1 has enzymatic activities of the methyltransferase (MTase) and the RNA-dependent RNA polymerase (RdRp), and plays a critical role in viral replication (19). Nevertheless, the precise roles of NSP1 in regulating host response remain a topic of ongoing investigation, with its implications for both viral pathogenesis and host defense yet to be fully elucidated.

Innate immune response, particularly the type I interferons (IFN-I), plays a crucial role in the host’s defense against viral infections (20). The signaling cascade mediated by IFN-I includes two key stages: IFN-I production and signal transduction. In the initial stage, the rapid production of IFN-I is triggered by the recognition of viral pathogen-associated molecular patterns (PAMPs) through host pattern recognition receptors (PRRs) (21). In the second stage, the secreted IFN-α/β engages with the IFN-I receptors (IFNARs) to activate JAK/STAT cascades. The activated STATs combine with interferon regulatory factor 9 (IRF9) to create the transcription complex of IFN-stimulated gene factor 3 (ISGF3). ISGF3 then translocates into the nucleus, where it binds to interferon-stimulated response elements (ISREs) and drives the expression of interferon-stimulated genes (ISGs) to suppress virus replication (22). It is well established that all known flaviviruses have evolved sophisticated strategies to antagonize the IFN-I response, enabling them to infect vertebrate hosts successfully. For example, the NS5 proteins of DENV and ZIKV are known to degrade STAT2 to inhibit the IFN-I response (23–26).

Here, we demonstrate that ALSV displays sensitivity to IFN-β and possesses the capability to counteract the IFN-I response through NSP1. In short, ALSV’s NSP1 selectively targets human STAT2 for degradation while having no effect on mouse STAT2. This degradation subsequently leads to a reduction in mitochondrial mass by disrupting mitochondrial dynamics to induce mitophagy and inhibiting mitochondrial biogenesis, resulting in the inhibition of innate immune responses.

## RESULTS

### ALSV infects multiple mammalian cell lines and induces an antiviral response

**Fig 1:**
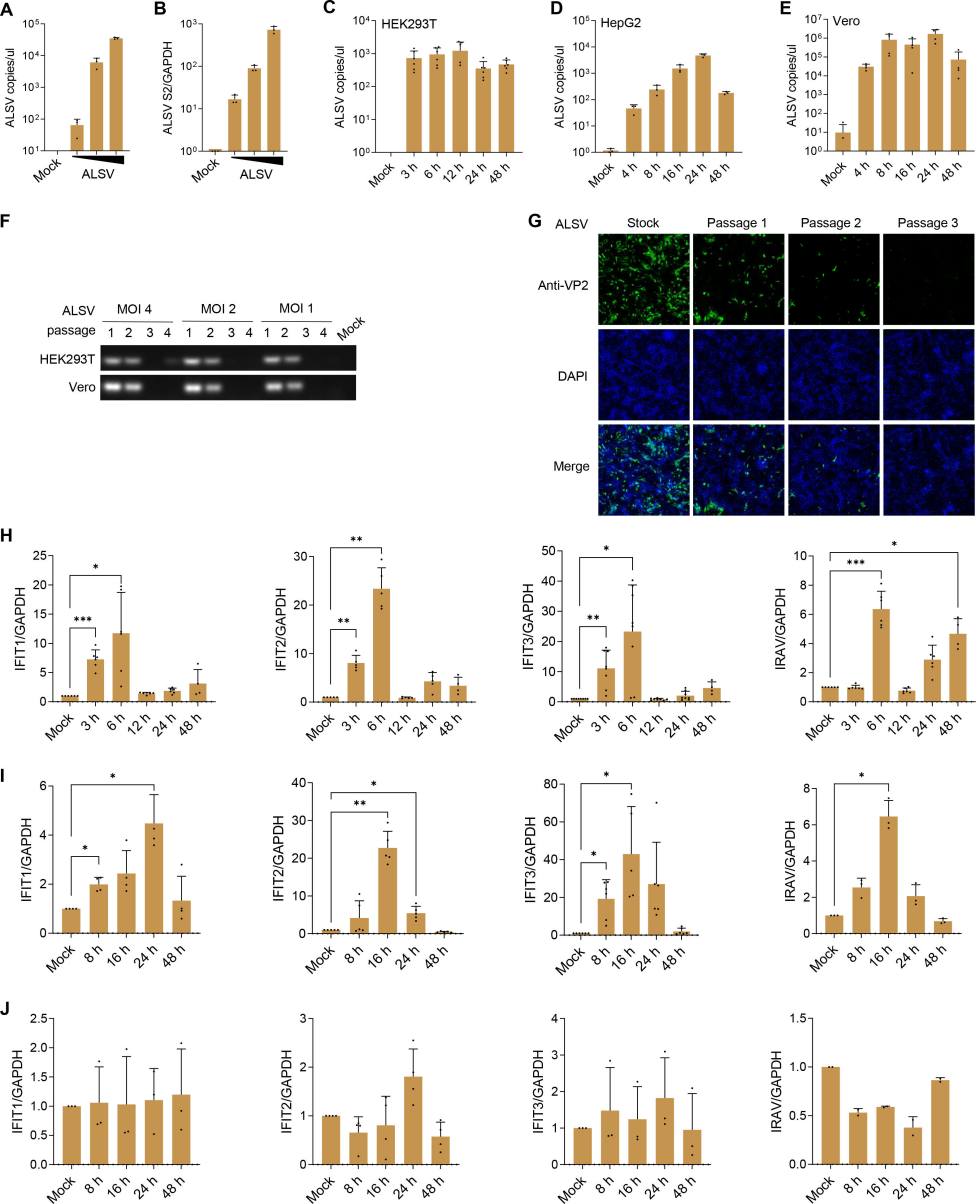
ALSV infection and host response in different cell types. (**A and B**) HEK293T cells were infected with escalating doses of ALSV (MOI 0.025, 0.25, and 2.5). At 48 hpi, viral RNA copies in supernatants were quantified using TaqMan-qPCR (**A**), and the mRNA levels of viral segment 2 (*S2*) relative to GAPDH control in cells were measured by qPCR (**B**). (**C–E**) HEK293T (**C**), HepG2 (**D**), and Vero (**E**) cells were infected with ALSV at MOI 2.5, the viral RNA copies in supernatants were assessed by TaqMan-qPCR at the indicated time points. (**F**) HEK293T and Vero cells were infected with escalating doses of ALSV (MOI 4, 2, and 1). After 2–3 days, the collected culture supernatant was used as the passage 1, which was then subcultured without dilution to passage 4. The viral replication ability in supernatants was assessed using RT-PCR and agarose gel electrophoresis. (**G**) ALSV was subcultured in Vero cells as indicated in (**F**). The collected viral supernatants were then infected with IRE/CVM19 tick cells. At 6 days post-infection, immunofluorescent staining was performed using anti-VP2 antibody. Nuclei were counterstained with DAPI. (**H–J**) HEK293T (**H**), HepG2 (**I**), and Vero (**J**) cells were infected with ALSV, and the mRNA levels of host *IFIT1*, *IFIT2*, *IFIT3*, and *IRAV* genes in cells were measured by qPCR at the indicated time points, with GAPDH serving as the internal reference control. Statistical analysis was performed using one-way ANOVA with multiple comparison correction (*P < 0.05, **P < 0.01, and ***P < 0.001).

As a newly discovered zoonotic pathogen, we investigated ALSV’s potential to infect and replicate in mammalian cells. HEK293T cells, derived from human embryonic kidney, were infected with escalating doses of ALSV grown in the tick cell line IRE/CTVM19 (8). At 48 hours post-infection (hpi), we examined viral replication levels, which showed that the ALSV copies in supernatants and the ALSV segment 2 (*S2*) mRNA level in cells increased with the escalating amount of infectious virus (Fig. 1A and B). In contrast, when HEK293T, HepG2, and Vero cells were infected with ALSV and incubated for the indicated times, the temporal replication dynamics revealed that the viral copies in supernatants and the *S2* mRNA levels in cells of HEK293T did not increase with the prolonged infection time (Fig. 1C; Fig. S1A); in HepG2 supernatants, the viral copies increased during the first 24 hpi and decreased at 48 hpi (Fig. 1D), while the *S2* mRNA levels remained unchanged over time (Fig. S1A), which is similar to that seen in Vero cells (Fig. 1E; Fig. S1A), suggesting that ALSV’s ability to replicate in mammalian cells may be limited. Then, we subcultured ALSV in both Vero and HEK293T cells and detected the viral genomic copies in culture supernatants, which showed that ALSV could be detected for the first two passages while becoming undetectable in the third passage (Fig. 1F; Fig. S1B). To confirm this result, we subcultured ALSV in Vero cells, and immunofluorescent analysis using VP2 antibody found that, compared to ALSV working stock, the infectivity of supernatants obtained from Vero cells significantly decreased with increasing passages, and no infectious viral particles were detected in the third-generation supernatant (Fig. 1G), suggesting that ALSV cannot be passaged more than twice in mammalian cells. Collectively, our findings indicate that ALSV can infect multiple mammalian cells but exhibits limited replication capability, which is consistent with the ability of JMTV (11, 27).

ALSV exhibits more robust replication in IFN-I-deficient Vero cells compared to HEK293T and HepG2 cells (Fig. 1C through E), suggesting that IFN-I may play a critical role in the host’s antiviral response. To gain insights into the interaction between ALSV and the IFN-I system, we firstly examined whether ALSV infection induces the expression of ISGs, and found that ALSV infection stimulated the expression of *IFIT1*, *IFIT2*, and *IFIT3* as early as 3 hpi and reached its peak at 6 hpi, while *IRAV* peaked at 6 hpi and then recovered at 24 and 48 hpi in HEK293T cells (Fig. 1H). In HepG2 cells, ALSV induced the expression of *IFIT1*, *IFIT2*, *IFIT3*, and *IRAV* as early as 8 hpi, with peaks at 16 or 24 hpi, which occurred later than that in HEK293T cells (Fig. 1I). This delay in ISG expression might explain the increased ALSV copies in HepG2 cells (Fig. 1C and D). Notably, ALSV did not stimulate ISG expression in Vero cells (Fig. 1J), which aligns with the fact that Vero cells are unable to produce IFN-I. These findings suggest that ALSV infection induces the expression of antiviral genes during the early stages of infection, contributing to the control of viral replication.

### ALSV exhibits sensitivity to IFN-β and antagonism to the type I IFN response

**Fig 2:**
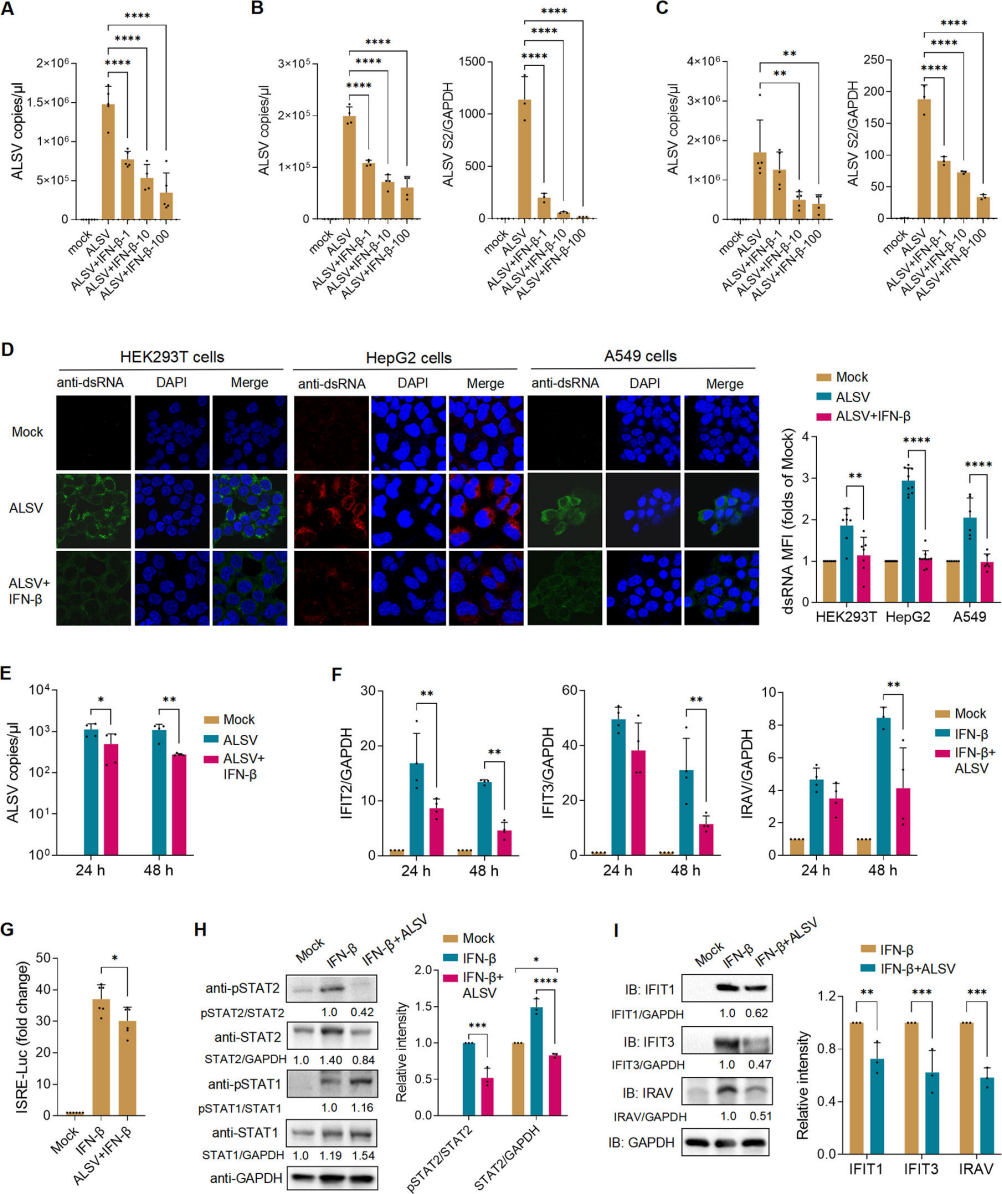
Sensitivity of ALSV to IFN-β and antagonism of type I IFN response. (**A–C**) HEK293T (**A**), HepG2 (**B**), and A549 (**C**) cells were infected with ALSV at MOI 2.5. At 2 hpi, cells were treated with increasing concentrations of IFN-β (1, 10, 100 ng/mL) for 24 hours. Viral copies in supernatant and the viral *S2* mRNA levels in cells were determined using qPCR. (**D**) HEK293T, HepG2, and A549 cells were infected with ALSV at MOI 2.5. At 2 hpi, cells were treated with IFN-β (10 ng/mL) for 24 hours and were subjected to immunofluorescence analysis using an anti-dsRNA antibody. Nuclei were counterstained with DAPI. The MFI per graph of dsRNA was analyzed (*n* ≥ 6 graphs). (**E**) HepG2 cells were infected with ALSV. At 2 hpi, cells were treated with or without IFN-β for 24 or 48 hours, and viral copies in supernatant were determined. (**F**) HepG2 cells treated with or without IFN-β were mock infected or infected with ALSV. At 24 or 48 hpi, the mRNA levels of host *IFIT2*, *IFIT3*, and *IRAV* genes were examined using qPCR, with GAPDH serving as the internal reference control. (**G**) HEK293T cells were co-transfected with an ISRE-luc plasmid and a control plasmid pGL4.74. At 24 hpt, cells were infected with ALSV and treated with IFN-β for 24 hours. Cells were harvested, and luciferase activity was measured. (**H and I**) HEK293T cells treated with IFN-β were mock infected or infected with ALSV. At 48 hpi, cells were analyzed by immunoblotting with the indicated antibodies. Grayscale statistical analyses of pSTAT2 relative to STAT2, STAT2 relative to GAPDH (**H**), and IFIT1, IFIT3, IRAV relative to GAPDH (**I**) are displayed on the right. Statistical analysis was performed using one- or two-way ANOVA with multiple comparison correction (*P < 0.05, **P < 0.01, ***P < 0.001, and ****P < 0.0001).

To investigate ALSV’s sensitivity to IFN-I, we infected various cells with ALSV and treated them with increasing concentrations of IFN-β for 24 hours. The virus quantification assays showed that IFN-β inhibited ALSV replication in a concentration-dependent manner (Fig. 2A through C). Immunofluorescence assay (IFA) of viral dsRNA further confirmed that IFN-β effectively suppressed ALSV replication (Fig. 2D); this assay, in which the anti-dsRNA antibody is used to detect viral dsRNA intermediates, has been successfully used to detect classical flaviviruses such as hepatitis C virus and DENV (28, 29). Moreover, we observed that longer durations of IFN-β treatment led to more effective inhibition of ALSV replication (Fig. 2E). Overall, these data underscore the sensitivity of ALSV to the action of IFN-β.

Efficient IFN-I antagonism is of particular importance for all vector-borne flaviviruses, and various strategies for evading the IFN response have been demonstrated in viruses such as DENV and ZIKV (25). To explore whether ALSV antagonizes the IFN-I response, we treated HepG2 cells with IFN-β and subsequently mock infected or infected them with ALSV. The qPCR results revealed that ALSV infection inhibited the expression of ISGs, including *IFIT2*, *IFIT3*, and *IRAV*, especially at 48 hpi (Fig. 2F). In HEK293T cells, we also found that ALSV infection suppressed IFN-β-induced ISRE promoter activity (Fig. 2G) and reduced the protein levels of phosphorylated STAT2 (Tyr689), and total STAT2, IFIT1, IFIT3, IRAV induced by IFN-β (Fig. 2H and I). These findings collectively demonstrated that ALSV, like other flaviviruses, has the capability to antagonize the IFN-I response.

### ALSV NSP1 antagonizes IFN-I response by degrading STAT2 through an autophagy pathway

**Fig 3:**
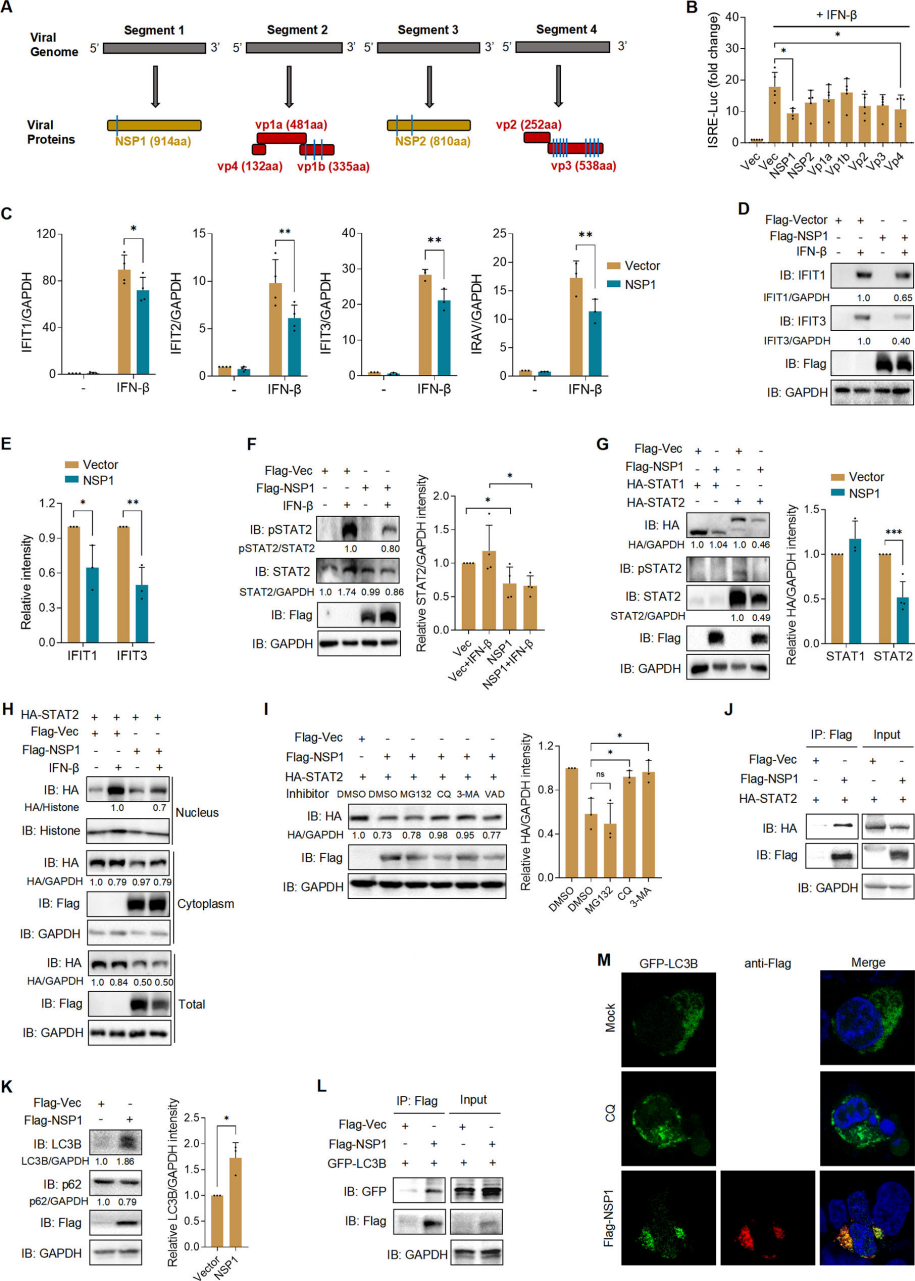
ALSV NSP1 protein antagonizes IFN-I signaling by mediating STAT2 degradation in an autophagy pathway. (**A**) Schematic diagrams of the ALSV genome, comprising four segments that encode structural and nonstructural proteins. The transmembrane region is indicated by the blue lines. (**B**) HEK293T cells were transfected with an ISRE reporter plasmid, a control plasmid, and plasmids expressing ALSV proteins. At 24 hpt, cells were treated with IFN-β for 12 hours, and luciferase activity was measured. (**C**) A549 cells cultured in 24-well plates were transfected with NSP1 or vector plasmid (0.25 µg/well). At 24 hpt, cells were treated with or without IFN-β for 12 hours, and the mRNA expression of *IFIT1*, *IFIT2*, *IFIT3*, and *IRAV* was examined using qPCR. (**D**) HEK293T cells were treated as in (**C**). At 24 hpt, cell lysates were analyzed by immunoblotting with anti-IFIT1, IFIT3, and Flag antibodies, with GAPDH as a loading control. (**E**) The grayscale statistical analysis of IFIT1 and IFIT3 relative to GAPDH of lines 2 and 4 in (**D**). (**F**) HEK293T cells were transfected with Flag-tagged NSP1 or vector. At 24 hpt, cells were treated with or without IFN-β for 12 hours, and cell lysates were analyzed by immunoblotting. Grayscale statistical analysis of STAT2 relative to GAPDH is displayed on the right. (**G**) HEK293T cells were co-transfected with NSP1 and STAT1 or STAT2 expressing plasmids. At 24 hpt, cell lysates were analyzed by immunoblotting. Grayscale statistical analysis of STAT1 and STAT2 relative to GAPDH is displayed on the right. (**H**) HEK293T cells were co-transfected with NSP1 and STAT2 plasmids. At 24 hpt, cells were treated with or without IFN-β for 30 minutes, then subjected to cell fractionation assay by immunoblotting. (**I**) HEK293T cells were co-transfected with NSP1 and STAT2 plasmids. At 24 hpt, cells were treated with the inhibitors MG132, CQ, 3-MA, and VAD. After 6 hours, cell lysates were analyzed by immunoblotting. Grayscale statistical analysis of STAT2 relative to GAPDH is displayed on the right. (**J**) HEK293T cells were co-transfected with HA-STAT2 together with NSP1 or vector plasmids. At 24 hpt, cells were treated with CQ for 6 hours, subjected to anti-Flag immunoprecipitates, and analyzed by immunoblotting. (**K**) HEK293T cells were transfected with NSP1 or vector plasmid. At 24 hpt, cell lysates were analyzed by immunoblotting with LC3B, p62, and Flag antibodies. Grayscale statistical analysis of LC3B relative to GAPDH is displayed on the right. (**L**) HEK293T cells were transfected with Flag-NSP1 and GFP-LC3B plasmids. At 48 hpt, anti-Flag immunoprecipitates were analyzed by immunoblotting. (**M**) GFP-LC3 dot formation in HepG2 cells transiently transfected with GFP-LC3B and either left untransfected (mock) or transfected with Flag-NSP1 for 48 hours or treated with CQ for 16 hours. Nuclei were counterstained with DAPI. Statistical analysis was conducted using one- or two-way ANOVA with multiple comparison correction (*P < 0.05, **P < 0.01, and ***P < 0.001).

To investigate which viral proteins of ALSV regulate IFN-β-induced downstream signaling, we subcloned ALSV genes into a Flag-tagged vector, encompassing nonstructural genes NSP1-2 and structural genes VP1a, VP1b, and VP2-4 (Fig. 3A) (18). We examined the individual effects of ALSV proteins on activation of the ISRE promoter, and found that NSP1 and VP4 significantly inhibited IFN-β-mediated ISRE promoter activation (Fig. 3B). To further confirm that NSP1 can inhibit IFN-I signaling, we transfected A549 cells with NSP1 or vector plasmid. At 24 hours post-transfection (hpt), cells were treated with IFN-β or mock treated for 12 hours. QPCR results demonstrated that NSP1 inhibited the mRNA levels of *IFIT1*, *IFIT2*, *IFIT3*, and *IRAV* (Fig. 3C). In HEK293T cells, we also observed that NSP1 suppressed IFN-β-induced ISG expression in a dose-dependent manner (Fig. S2A) and reduced the protein levels of IFIT1 and IFIT3 induced by IFN-β (Fig. 3D and E). These findings provide strong evidence that ALSV NSP1 inhibits the expression of ISGs induced by IFN-β.

We then delved into the mechanism through which NSP1 inhibits IFN-I signaling. To investigate whether NSP1-mediated IFN-I antagonism functions upstream or downstream of transcription factors STAT1/STAT2, we assessed the protein levels and activity of STAT1/2 in HEK293T cells expressing NSP1. Immunoblotting revealed that NSP1 reduced the expression and phosphorylation levels of STAT2 induced by IFN-β, regardless of the presence or absence of IFN-β (Fig. 3F and G). However, it did not affect total or phosphorylated STAT1 (Fig. S2B). In addition, measurement of protein extracted from the nucleus and cytoplasm and IFA analysis showed that the expression of NSP1 prevented IFN-β-mediated nuclear translocation of STAT2 (Fig. 3H; Fig. S2C). Taken together, our findings reveal that ALSV NSP1 antagonizes IFN-I signaling by targeting STAT2.

The reduction of STAT2 induced by NSP1 may result from the inhibition of protein synthesis or the promotion of protein degradation. Both luciferase assay and qPCR results revealed that NSP1 did not inhibit STAT2 promoter activity or mRNA expression, which were consistent with those observed for STAT1 (Fig. S2D and E), demonstrating that NSP1 did not affect STAT2 synthesis. Then, we explored whether NSP1 induces STAT2 degradation through specific pathways. HEK293T cells were co-transfected with NSP1 and STAT2 plasmids, followed by treatment with dimethyl sulfoxide (DMSO), the proteasome inhibitor MG132, the autophagy inhibitors CQ and 3-MA, or the pan-caspase inhibitor Z-VAD-FMK, which revealed that CQ and 3-MA reversed NSP1-induced STAT2 reduction (Fig. 3I), indicating that NSP1 degraded STAT2 through an autophagy pathway. Furthermore, the co-immunoprecipitation (Co-IP) results showed that NSP1 bound to STAT2, especially in the presence of CQ, and this binding was independent of IFN-β treatment (Fig. 3J; Fig. S2F). We also found that NSP1 induced the expression of autophagosome maker LC3B and increased the LC3-II/LC3-I ratio, while reducing the p62 level (Fig. 3K), and interacted with LC3B (Fig. 3L). Enhanced autophagic flux was further validated by detecting an increase in GFP-LC3-positive autophagosome formation induced by NSP1, and the co-localization of NSP1 with LC3B and p62 (Fig. 3M; Fig. S2G). These results collectively demonstrate that NSP1 degrades STAT2 in an autophagy-dependent pathway.

### ALSV NSP1 reduces mitochondrial mass by disrupting mitochondrial dynamics to induce mitophagy and inhibiting biogenesis

**Fig 4:**
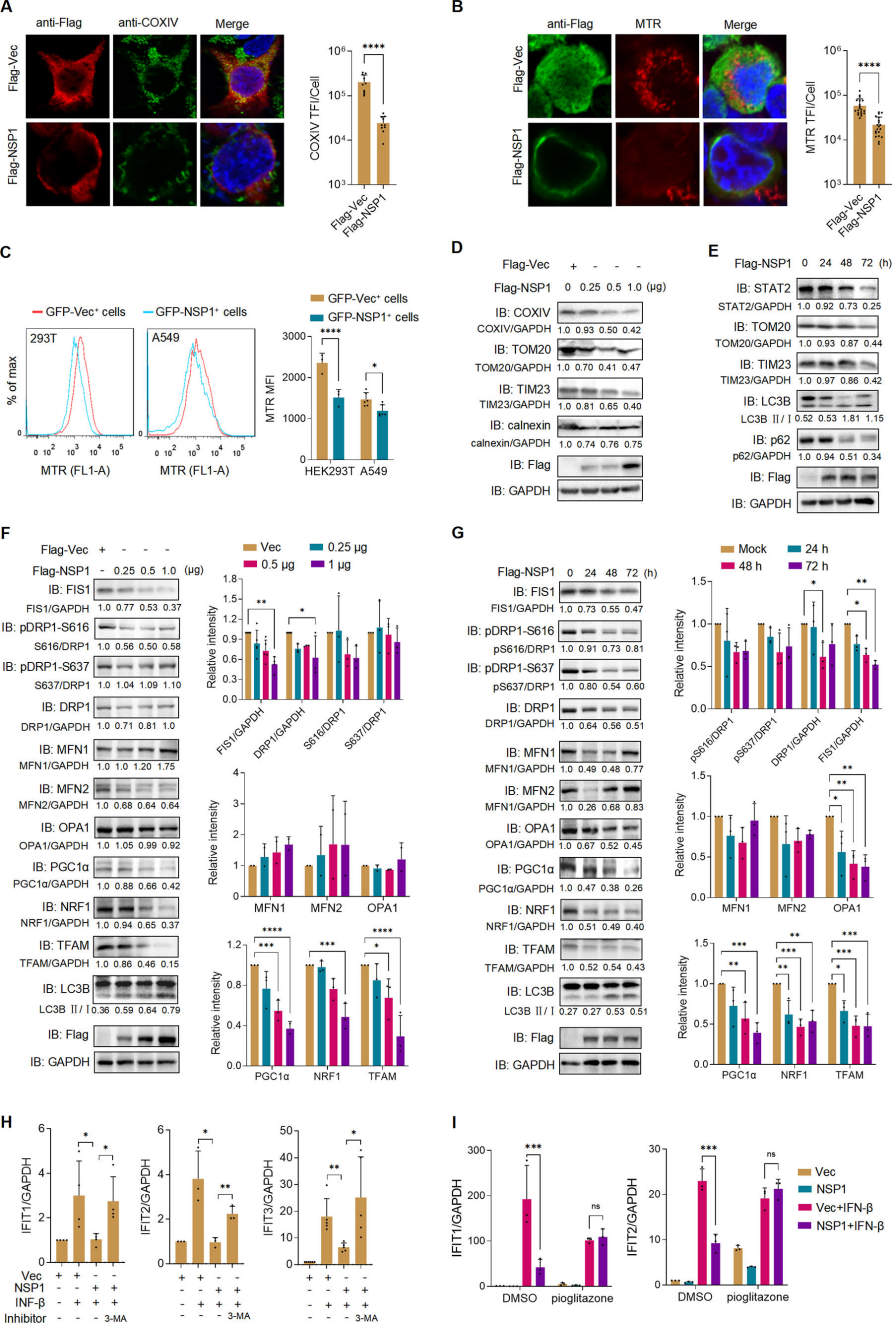
Alongshan virus NSP1 reduces mitochondrial mass by disrupting mitochondrial dynamics to induce mitophagy and inhibiting its biogenesis. (**A and B**) HEK293T cells were transfected with Flag-tagged NSP1 or vector plasmid. At 24 hpt, cells were subjected to immunofluorescence with anti-Flag and COXIV antibodies (**A**) or stained with MTR (**B**). The TFI of COXIV or MTR per cell was analyzed (*n* ≥ 10 cells). (**C**) HEK293T or A549 cells were transfected with NSP1 with an unfused GFP tag. At 48 hpt, cells were stained with MTR and detected by flow cytometry. The MFI of MTR was analyzed in GFP^+^ cells (*n* ≥ 3). (**D**) HEK293T cells were transfected with escalating doses of NSP1 plasmid. At 24 hpt, cell lysates were analyzed by immunoblotting with the indicated antibodies. (**E**) HEK293T cells were transfected with NSP1 and collected at the indicated times. Cell lysates were analyzed by immunoblotting. (**F**) HEK293T cells were transfected with escalating doses of NSP1. At 24 hpt, cell lysates were analyzed by immunoblotting. Grayscale statistical analysis is displayed on the right. (**G**) HEK293T cells were transfected with NSP1 and collected at the indicated times. Cell lysates were analyzed by immunoblotting. Grayscale statistical analysis is displayed on the right. (**H**) HEK293T cells were transfected with NSP1 or vector. At 24 hpt, cells were treated with or without IFN-β and 3-MA for 12 hours, and the mRNA levels of *IFIT1*, *IFIT2*, and *IFIT3* were measured by qRT-PCR. (**I**) HEK293T cells were transfected with NSP1 or vector. At 24 hpt, cells were treated with or without IFN-β and pioglitazone (1 µM) for 16 hours, and the mRNA levels of *IFIT1* and *IFIT2* were measured by qRT-PCR. Statistical analysis was conducted using one- or two-way ANOVA with multiple comparison correction (*P < 0.05, **P < 0.01, ***P < 0.001, and ****P < 0.0001).

The membrane (M) protein of severe acute respiratory syndrome coronavirus 2 (SARS-CoV-2) has been shown to reduce mitochondrial mass by inducing mitophagy to suppress the IFN-I response, while lipopolysaccharide (LPS) increases mitochondrial mass to promote inflammatory cytokine production, which suggests that mitochondrial mass plays a critical role in the immune response (30, 31). Given that NSP1 reduces the mitochondrial mass (Fig. 4A) (18), we hypothesized that NSP1-induced reduction in mitochondrial mass may be related to IFN-I antagonism. To assess mitochondrial mass, we used MitoTracker Red (MTR), a fluorescent probe independent of mitochondrial Δψm. We conducted IFA and flow cytometry analysis to measure the MTR fluorescence intensity in various cells expressing Flag- or GFP-tagged NSP1, which consistently demonstrated that NSP1 reduced mitochondrial mass (Fig. 4B and C; Fig. S3A and B). Furthermore, NSP1 dose-dependently reduced the levels of mitochondrial marker proteins, such as COXIV, TOM20, and TIM23, while not affecting the endoplasmic reticulum marker calnexin (Fig. 4D). Additionally, NSP1 led to a time-dependent decrease of STAT2, TOM20, and TIM23, accompanied by an increase in the LC3-II/LC3-I ratio and a decrease in p62 (Fig. 4E). These findings collectively confirm that NSP1 induces mitochondrial mass reduction and mitophagy.

The regulation of mitochondrial mass, which encompasses mitochondrial dynamics, mitophagy, and mitochondrial biogenesis, constitutes a pivotal mechanism for coordinating the structure and function of mitochondria (32). Mitochondrial dynamics is the essential physiological process of mitochondrial fusion and fission. Mitochondrial fusion can facilitate the rapid exchange of metabolites and complement impaired mitochondria to enhance their functionality, and is controlled by the outer membrane fusion proteins, mitofusins 1 and 2 (MFN1 and 2), and the inner membrane fusion protein, optic atrophy 1 (OPA1) (33, 34). On the other hand, mitochondrial fission involves the division of a mitochondrion into two new organelles and is mediated by conserved dynamin family GTPases: mitochondrial fission 1 protein (FIS1) and dynamin-related protein 1 (DRP1) (34). Subsequently, damaged mitochondria induced by imbalance in mitochondrial dynamics are targeted for degradation through mitophagy, a specific autophagic process responsible for eliminating dysfunctional mitochondria (35). To elucidate the underlying mechanisms through which ALSV NSP1 reduces mitochondrial mass, we investigated the expression of proteins involved in mitochondrial dynamics and found that NSP1 reduced the levels of fission-related proteins FIS1 and DRP1 in a dose-dependent manner, without affecting DRP1 phosphorylation or the fusion-related proteins (Fig. 4F). Furthermore, NSP1 decreased the levels of FIS1, DRP1, and OPA1 in a time-dependent manner, while other mitochondrial dynamics-related proteins remained unaffected (Fig. 4G). These results suggest that NSP1 induces mitophagy via disrupting mitochondrial dynamics.

A reduced mitochondrial population can trigger mitochondrial biogenesis, a process that is controlled by transcriptional factors such as peroxisome proliferator activator receptor gamma-coactivator 1α (PGC-1α), nuclear respiratory factor 1 (NRF1), and mitochondrial transcription factor A (TFAM). Previous studies have shown that STAT2 promotes LPS-induced mitochondrial mass increase through the transcriptional activation of mitochondrial biogenesis (31). Given that NSP1 can degrade STAT2 (Fig. 3), we speculated that NSP1 might inhibit mitochondrial biogenesis. As expected, our experiments revealed that NSP1 indeed inhibited the expression of PGC-1α, NRF1, and TFAM (Fig. 4F and G). Peroxisome proliferator-activated receptors-γ (PPARγ) is a ligand-activated transcription factor that plays a role in regulating PGC1α expression (36), and the PPARγ agonists such as pioglitazone or rosiglitazone have been shown to stimulate mitochondrial biogenesis and enhance mitochondrial function in conditions such as Alzheimer’s disease, Parkinson’s disease, and Huntington’s disease (37, 38). We further investigated the relationship between NSP1-mediated mitochondrial mass reduction and IFN-I antagonism, and found that the mitophagy-specific inhibitor 3-MA and the PPARγ agonist pioglitazone could reverse NSP1-mediated inhibition of ISGs (Fig. 4H and I). These results suggest that the induction of mitophagy and the inhibition of mitochondrial biogenesis contribute to NSP1-induced mitochondrial mass reduction and IFN-I antagonism.

### ALSV reduces mitochondrial mass in a STAT2-dependent manner

**Fig 5:**
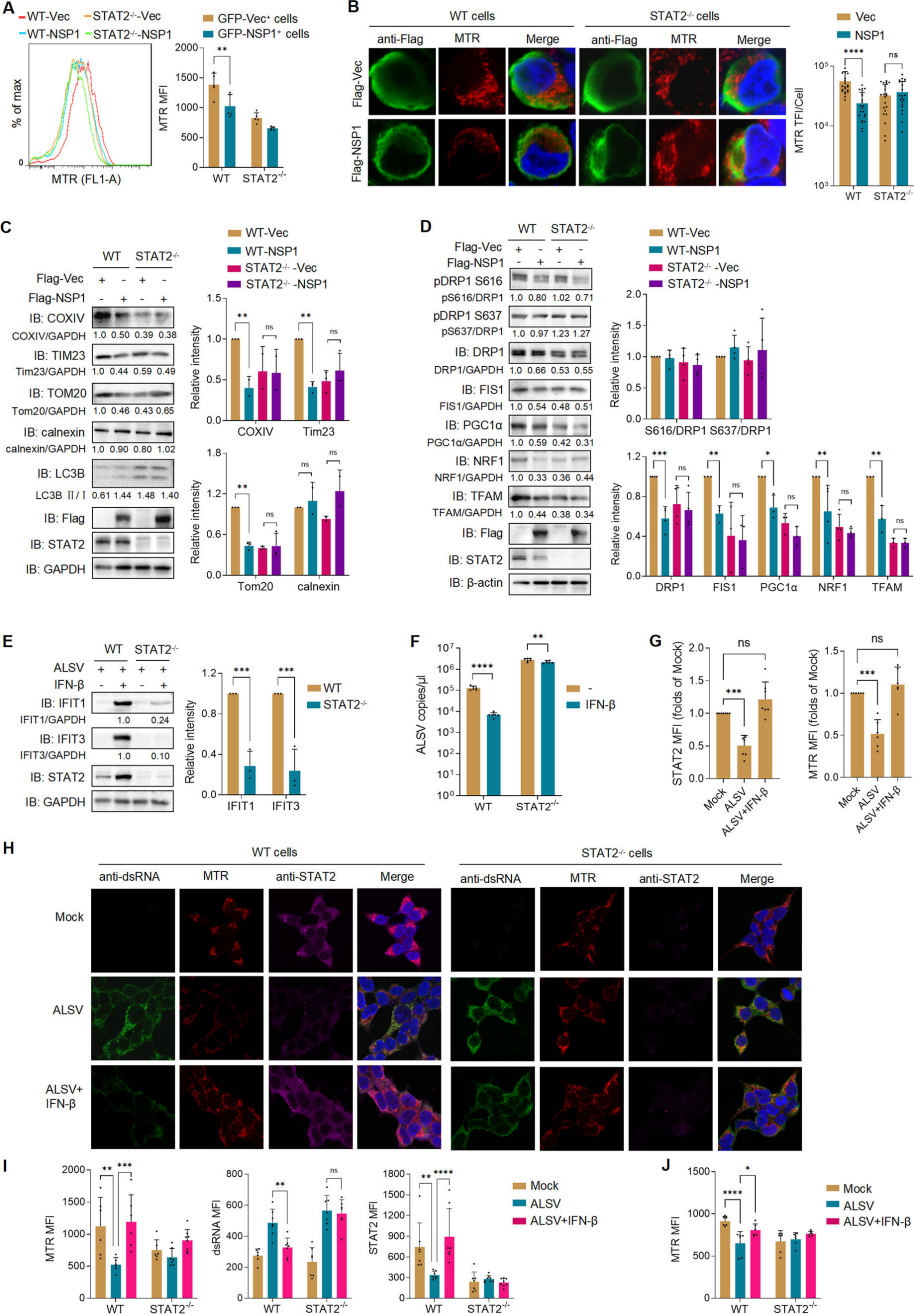
ALSV reduces mitochondrial mass in a STAT2-dependent manner. (**A**) HEK293T WT or STAT2 knockout (STAT2^-/-^) cells were transfected with ALSV NSP1 with an unfused GFP tag. At 48 hpt, cells were stained with MTR and analyzed by flow cytometry. The MFI of MTR was measured in GFP^+^ cells (*n* = 5). (**B**) WT or STAT2^-/-^ cells were transfected with NSP1 or vector plasmid. At 48 hpt, cells were stained with MTR and anti-Flag antibody. The TFI of MTR per cell was measured (*n* ≥ 20 cells). (**C and D**) WT or STAT2^-/-^ cells were transfected with Flag-NSP1 or vector. At 24 hpt, cell lysates were analyzed by immunoblotting with the indicated antibodies. Grayscale statistical analysis is displayed on the right. (**E and F**) WT or STAT2^-/-^ cells were infected with ALSV at MOI 2.5. At 2 hpi, cells were treated with or without IFN-β for 24 hours. Cell lysates were analyzed by immunoblotting. Grayscale statistical analysis of IFIT1 and IFIT3 relative to GAPDH control in lines 2 and 4 is displayed on the right (**E**). The viral RNA copies in supernatants were determined using TaqMan-qPCR (**F**). (**G**) A549 cells were infected with ALSV. At 2 hpi, cells were treated with or without IFN-β for 24 hours. The cells were stained with anti-dsRNA and STAT2 antibodies. The MFI of STAT2 and MTR per image was measured (*n* ≥ 5 images). (**H**) HEK293T WT or STAT2^-/-^ cells were infected with ALSV. At 2 hpi, cells were treated with or without IFN-β. Cells were stained with anti-dsRNA and STAT2 antibodies along with MTR. (**I**) The MFI of MTR, dsRNA, and STAT2 per image indicated in (**H**) was analyzed (*n* ≥ 6 images). (**J**) WT or STAT2^-/-^ cells were infected with ALSV. At 2 hpi, cells were treated with IFN-β and stained with MTR, then detected by flow cytometry. The MTR MFI was analyzed (*n* = 4). Statistical analysis was performed using one- or two-way ANOVA with multiple comparison correction (*P < 0.05, **P < 0.01, ***P < 0.001, and ****P < 0.0001).

Recent studies have highlighted the pivotal role of STAT2 in maintaining mitochondrial homeostasis by promoting mitochondrial fission and biogenesis (31, 39, 40). To explore whether NSP1 reduces mitochondrial mass in a STAT2-dependent manner, we knocked out the STAT2 gene in HEK293T cells (Fig. S4A). Consistent with previous studies, we observed a reduction in mitochondrial mass in STAT2^-/-^ cells (Fig. S4B). We transfected GFP-tagged NSP1 or vector plasmids into wild-type (WT) and STAT2^-/-^ cells. After 48 hours, cells were stained with MitoTracker Red CMXRos (MTR, YEASEN, Cat# 40741ES50), a mitochondrial dye that is independent of mitochondrial membrane potential (Δψm) and is used to monitor mitochondria, and analyzed by flow cytometry. The mean fluorescence intensity (MFI) of GFP-positive cells indicated that NSP1 was no longer able to reduce mitochondrial mass in STAT2^-/-^ cells (Fig. 5A). Similarly, mitochondrial mass was not decreased in STAT2^-/-^ cells infected with lentiviral particles expressing GFP-NSP1 (Fig. S4C). IFA results confirmed that the absence of STAT2 rendered NSP1 unable to further reduce mitochondrial mass (Fig. 5B). Furthermore, immunoblotting results showed that NSP1 did not reduce the levels of mitochondrial proteins COXIV, TOM20, or TIM23, nor increased the ratio of LC3-II/LC3-I in STAT2^-/-^ cells, suggesting that NSP1 cannot induce stronger mitophagy without STAT2 (Fig. 5C). Additionally, in STAT2^-/-^ cells, NSP1 did not lead to further reduction of proteins associated with mitochondrial dynamics (FIS1 and DRP1) or mitochondrial biogenesis (PGC1α, NRF1, and TFAM) (Fig. 5D). These findings collectively suggested that ALSV NSP1 relies on STAT2 to reduce mitochondrial mass.

Our observations have revealed that ALSV infection suppresses both total STAT2 protein levels and its phosphorylation (Fig. 2H). In STAT2^-/-^ cells, the ability of IFN-β to induce the expression of ISGs and inhibit ALSV replication is abrogated (Fig. 5E and F), strongly suggesting that ALSV inhibits IFN-I signaling through its specific targeting of STAT2. Consequently, we proceeded to investigate whether ALSV reduces the mitochondrial mass in a manner dependent on STAT2. Our initial findings indicated that ALSV infection led to a decrease in mitochondrial mass, and this reduction was effectively reversed by IFN-β (Fig. 5G; Fig. S4D), highlighting the role of IFN-β in mitigating the virus-induced decline in mitochondrial mass. Furthermore, the knockout of STAT2 abolished the inhibitory effect of IFN-β on ALSV replication, and IFN-β no longer counteracted the ALSV-induced loss of mitochondrial mass in the absence of STAT2 (Fig. 5H and I). These findings were further corroborated by flow cytometry data, which confirmed that IFN-β failed to eliminate ALSV-mediated reduction in mitochondrial mass in STAT2^-/-^ cells (Fig. 5J). These data collectively elucidate that ALSV infection also relies on STAT2 to mediate the reduction in mitochondrial mass.

### Mapping the interaction between ALSV NSP1 and STAT2

**Fig 6:**
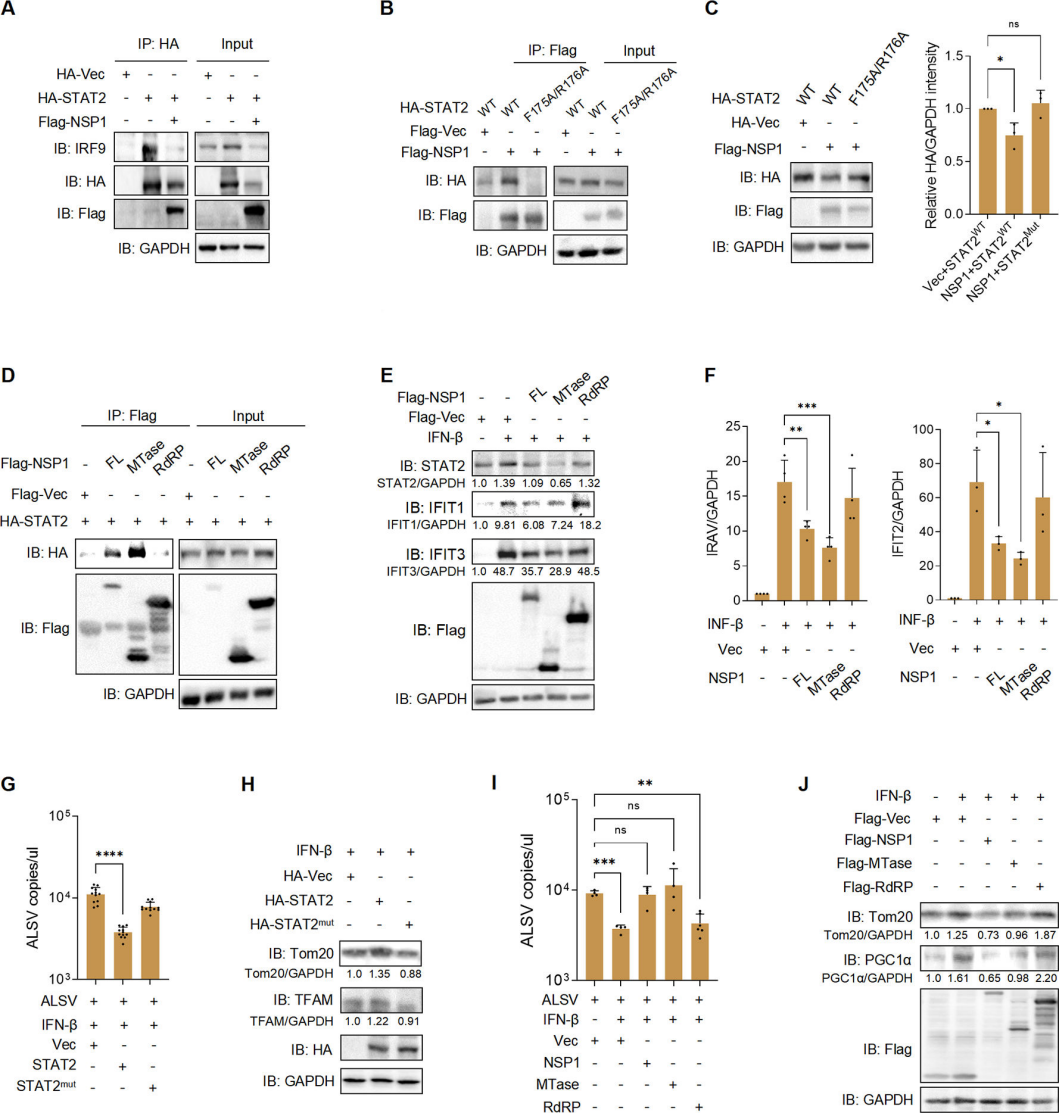
Mapping the interaction between ALSV NSP1 and STAT2. (**A**) HEK293T cells were transfected with HA-STAT2 with or without Flag-NSP1. At 48 hpt, anti-HA immunoprecipitates were analyzed by immunoblotting. (**B**) HEK293T cells were transfected with NSP1 or vector along with STAT2 WT or F175A/R176B mutant. At 48 hpt, anti-Flag immunoprecipitates were analyzed by immunoblotting. (**C**) HEK293T cells were transfected with NSP1 or vector along with STAT2 WT or F175A/R176B mutant. At 24 hpt, cell lysates were analyzed by immunoblotting. Grayscale statistical analysis of HA relative to GAPDH is displayed on the right. (**D**) HEK293T cells were transfected with STAT2 and the indicated truncations of NSP1. At 48 hpt, anti-Flag immunoprecipitates were analyzed by immunoblotting. (**E and F**) HEK293T cells were transfected with plasmids expressing truncations of NSP1 or vector. At 24 hpt, cells were treated with or without IFN-β for 12 hours. Cell lysates were analyzed by immunoblotting (**E**), and the mRNA expression of *IRAV* and *IFIT2* was examined using qPCR (**F**). (**G and H**) STAT2^-/-^ cells were transfected with STAT2 WT or F175A/R176B mutant. At 24 hpt, cells were infected with ALSV and treated with IFN-β for 24 hours. The viral RNA copies in supernatants were determined using TaqMan-qPCR (**G**), and cell lysates were analyzed by immunoblotting (**H**). (**I and J**) HEK293T cells were transfected with the indicated truncations of NSP1. At 24 hpt, cells were infected with ALSV and treated with IFN-β for 24 hours. The viral RNA copies in supernatants were determined (**I**), and cell lysates were analyzed by immunoblotting (**J**). Statistical analysis was conducted using one-way ANOVA with multiple comparison correction (*P < 0.05, **P < 0.01, ***P < 0.001, and ****P < 0.0001).

The structural basis for the suppression of human STAT2 by flavivirus NS5 has been elucidated, revealing a multifaceted interaction between STAT2 and NS5 of ZIKV and DENV. One facet involves the MTase and RdRp domains of NS5 forming a conserved interdomain cleft to prevent the interaction between STAT2 and IRF9 (41). In our study, we aimed to examine whether ALSV NSP1 similarly affects the association of STAT2 with IRF9. Co-IP revealed that NSP1 effectively reduces the binding of STAT2 to IRF9 (Fig. 6A). Another significant interaction involves NS5 binding to key sites F175/R176 located in the coiled-coil domain (CCD) of STAT2 (41). This finding raised the intriguing possibility that ALSV NSP1 might interact with STAT2 in a similar manner. As anticipated, our experiments demonstrated that the STAT2 F175A/R176A mutation substantially diminishes the binding of STAT2 to NSP1 (Fig. 6B). Furthermore, this mutation prevents STAT2 from undergoing degradation by NSP1 (Fig. 6C), providing compelling evidence that ALSV NSP1 indeed binds to STAT2 through the F175/R176 sites, ultimately leading to STAT2 degradation.

In the context of ZIKV and DENV NS5, the key sites for interaction with STAT2 have been identified as D734/H855 and D732/L853, respectively (41). Given that ALSV NSP1 shares only about 30% amino acid sequence homology with NS5, and notably, the key sites where NS5 binds to STAT2 are not conserved in NSP1 (Fig. S5), we embarked on an investigation to pinpoint which domain of NSP1 is involved in its interaction with STAT2. Remarkably, our findings revealed that the NSP1 MTase domain, but not the RdRp domain of NSP1, is responsible for interacting with STAT2 (Fig. 6D). Intriguingly, the MTase domain alone is sufficient to induce STAT2 degradation and suppress ISG expression (Fig. 6E and F). Furthermore, we transfected STAT2 WT or mutant into STAT2^-/-^ cells, showing that IFN-β was no longer able to inhibit ALSV replication in the presence of STAT2 mutant (Fig. 6G). Additionally, the expression of the STAT2 mutant in STAT2^-/-^ cells did not lead to an increase in mitochondrial mass (Fig. 6H).

Lastly, our investigation aimed to determine which domain of NSP1 is responsible for promoting ALSV replication by inhibiting IFN-β signaling. Our results conclusively demonstrated that only MTase of NSP1 promoted viral replication and reduced mitochondrial mass (Fig. 6I and J), providing further substantiation that the MTase domain plays a crucial role in antagonism of the IFN-I response by NSP1. In summary, our study elucidates that ALSV NSP1 disrupts the association of STAT2 and IRF9, leading to the inhibition of ISG expression. Moreover, the MTase domain of NSP1 emerges as a key player in this process, shedding light on the intricate mechanisms underlying ALSV evasion of the host immune response.

### ALSV NSP1 exhibits species-specific antagonism of STAT2

**Fig 7:**
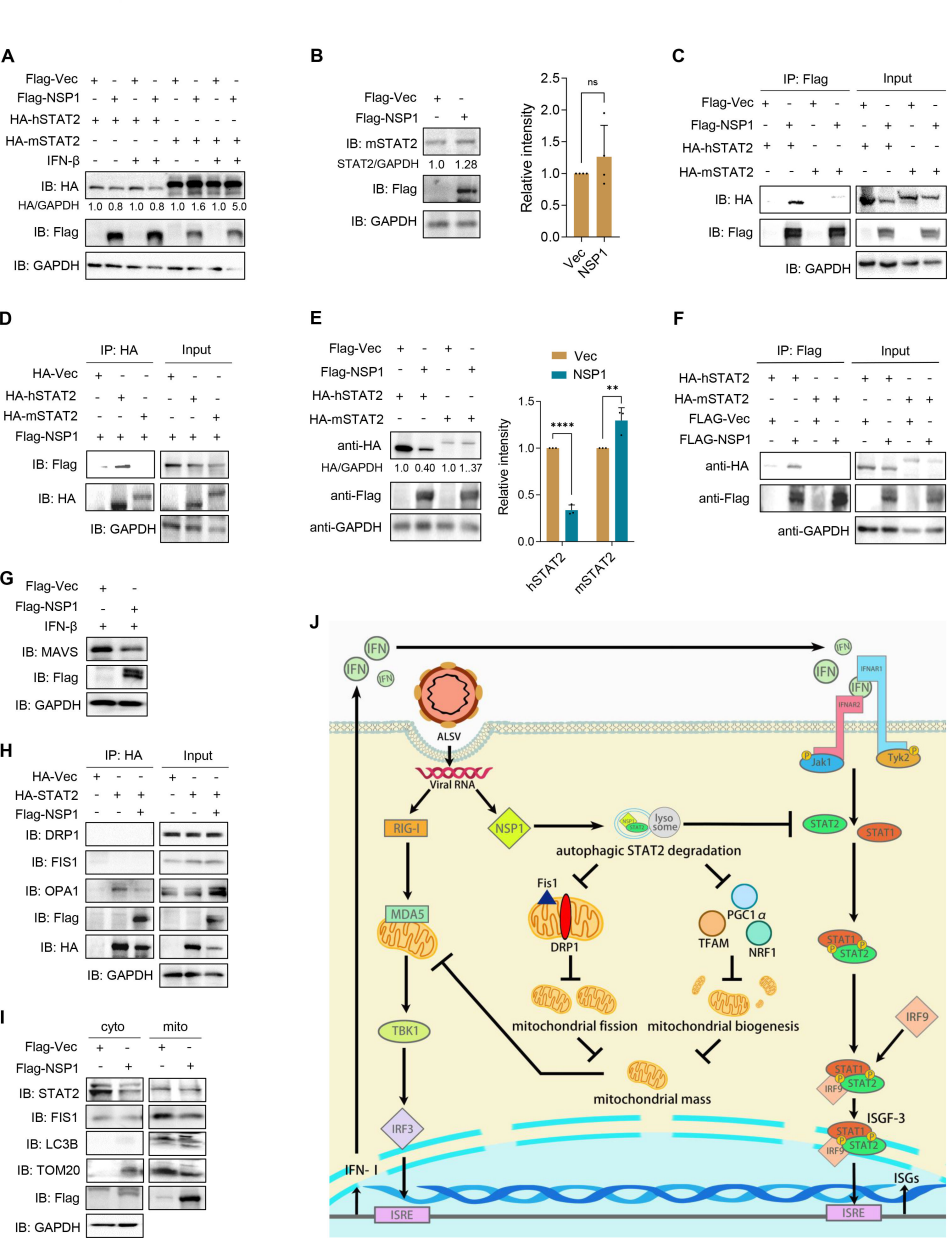
ALSV NSP1 exhibits species-specific antagonism of STAT2. (**A**) HEK293T cells were transfected with NSP1 or vector, along with hSTAT2 or mSTAT2. At 24 hpt, cells were treated with or without IFN-β for 12 hours, and cell lysates were analyzed by immunoblotting. (**B**) RAW264.7 cells were co-transfected with NSP1 or vector plasmid. At 24 hpt, cell lysates were analyzed by immunoblotting. Grayscale statistical analysis of mSTAT2 relative to GAPDH is displayed on the right. (**C and D**) HEK293T cells were transfected with Flag-tagged NSP1 or vector, along with HA-tagged hSTAT2 or mSTAT2. At 48 hpt, anti-Flag (**C**) or anti-HA (**D**) immunoprecipitates were analyzed by immunoblotting. (**E and F**) STAT2^-/-^ cells were transfected with hSTAT2 or mSTAT2, along with NSP1 or vector. At 48 hpt, cell lysates were analyzed by immunoblotting, and the grayscale statistical analysis of HA relative to GAPDH is displayed on the right (**E**). Moreover, anti-Flag immunoprecipitates were analyzed (**F**). (**G**) HEK293T cells were transfected with Flag-tagged NSP1 or vector plasmid. At 24 hpt, cells were treated with or without IFN-β for 12 hours, and cell lysates were analyzed by immunoblotting. (**H**) HEK293T cells were transfected with HA-STAT2 with or without Flag-NSP1. At 48 hpt, anti-HA immunoprecipitates were analyzed by immunoblotting with DRP1, FIS1, and OPA1 antibodies. (**I**) HEK293T cells were transfected with Flag-tagged NSP1 or vector plasmid. At 48 hpt, mitochondria were isolated, and cytoplasmic (cyto) and mitochondrial (mito) proteins were measured by immunoblotting with the indicated antibodies. (**J**) Schematic representation of the mechanisms underlying ALSV reduces mitochondrial mass via degrading STAT2 to suppress the innate immune response. Statistical analysis was conducted using one- or two-way ANOVA with multiple comparison correction (**P < 0.01 and ****P < 0.0001).

Previous studies have demonstrated that the ability of NS5 from DENV and ZIKV to interact with and degrade STAT2 is species specific, particularly affecting human STAT2 (hSTAT2) but not mouse STAT2 (mSTAT2) (26). This specificity underscores the importance of NS5-mediated IFN antagonism for efficient virus replication. In this context, we were keen to investigate whether ALSV NSP1 could target mSTAT2 for degradation, and found that NSP1 did not reduce the levels of exogenous and endogenous mSTAT2 (Fig. 7A and B). Furthermore, we explored the interaction between NSP1 and mSTAT2 by performing Co-IPs, showing that NSP1 bound to hSTAT2 but not to mSTAT2 (Fig. 7C), and vice versa (Fig. 7D). Upon re-expressing hSTAT2 or mSTAT2 in STAT2^-/-^ cells, we observed that compared to hSTAT2, NSP1 did not degrade and co-precipitate with mSTAT2 (Fig. 7E and F). These results conclusively establish that ALSV NSP1 is unable to target mSTAT2 for degradation.

## DISCUSSION

Understanding the pathogenic mechanisms of emerging segmented flavivirus serves as a foundational basis for the development of therapeutic drugs. In this study, we found that ALSV is sensitive to IFN-β and possesses the capacity to counteract the IFN-I response. Mechanistically, ALSV NSP1 selectively binds to and degrades human STAT2, effectively disrupting the IFN-I downstream signaling pathway. Furthermore, it results in a reduction in mitochondrial mass through perturbing mitochondrial dynamics to induce mitophagy and inhibiting its biogenesis, ultimately leading to the suppression of the innate immune response (Fig. 7J).

The intricate interplay between virus–host interactions that govern induction and evasion of the IFN-I response resembles a complex dance, aiming to achieve an optimal balance between virus replication, host disease, and host survival (42). Flavivirus infections are known to trigger the innate immune response by engaging PRRs, subsequently leading to upregulation of immune-related gene transcripts and serum IFNs (43, 44). Here, we discovered that ALSV can infect multiple mammalian cell lines and triggers the expression of antiviral genes that play a pivotal role in exerting control over viral replication.

On the other hand, viruses must effectively subvert the host’s IFN-I response in order to replicate or spread to new hosts. In the realm of flaviviruses, various viral nonstructural proteins have been shown to interfere with the IFN-I response, either by degrading or inhibiting host immune proteins (45–48). Among these, flavivirus NS5 possesses the highest potency and specificity as an IFN antagonist, albeit through virus-specific mechanisms (25). WNV NS5 impedes the surface expression of IFNAR1 (49); YFV NS5 relies on IFN treatment to interact with STAT2, thereby preventing STAT2 from binding to the ISRE promoter (50); DENV NS5 engages the E3 ubiquitin ligase UBR4 to degrade STAT2; ZIKV NS5 targets STAT2 for proteasomal degradation independently of UBR4 (24, 25). In our study, we unveiled that ALSV NSP1 utilizes autophagy pathway to degrade human STAT2, which is different from other flaviviruses.

Unlike other STAT family members, STAT2 shows a lower degree of homology between humans and mice (51, 52), which may explain the inability of NS5 to degrade murine STAT2. Structural analyses of canonical flavivirus NS5-STAT2 complexes have revealed that NS5 MTase and RdRp domains establish a conserved inter-domain cleft that impedes STAT2 association with IRF9. In line with this, our study also found that segmented flavivirus NSP1 binds to STAT2 to inhibit its interaction with IRF9, leading to the inhibition of ISGs. Intriguingly, unlike the NS5 RdRp domain interaction with STAT2, the NSP1 MTase domain is responsible for binding to STAT2. This may be caused by the structural differences between the NSP1 and NS5 MTase domains, which are unique to segmented flaviviruses (19). However, whether NSP1-induced degradation of STAT2 necessitates the presence of the E3 ligase UBR4 remains a subject for further study.

The pivotal link between mitochondria and the innate immune response was initially brought to light with the discovery of MAVS, an integral protein localized within mitochondria that plays a crucial role as an adaptor in RIG-I-like receptor signaling (53). More recently, other innate immune molecules, such as cGAS, NLRX1, TRAF6, NLRP3, and IRGM, have been functionally associated with mitochondria (54, 55). The accumulating evidence suggests that viruses eliminate critical immune molecules within mitochondria by reducing mitochondrial mass to evade the immune response (56, 57). For instance, the influenza A virus PB1-F2 protein degrades MAVS by triggering mitophagy (58); coronaviruses, including SARS-CoV-2, promote their replication by altering mitochondrial dynamics and targeting MAVS (57, 59); ZIKV NS4A induces mitochondrial fission and mitophagy to suppress the MAVS-mediated IFN-I response (60); DENV disrupts mitochondrial biogenesis by downregulating the master regulators PPARγ and PGC1α (61). In the present study, we uncovered that ALSV NSP1 disrupts mitochondrial dynamics by reducing the levels of the fission-related proteins FIS1 and DRP1 to induce mitophagy, and inhibits mitochondrial biogenesis through suppressing the expression of PGC-1α, NRF1, and TFAM, ultimately resulting in a decrease in mitochondrial mass. Moreover, NSP1 eliminated MAVS within mitochondria by diminishing mitochondrial mass, consequently inhibiting IFN-I production (Fig. 7G). Interestingly, promoting mitochondrial mass through inhibiting mitophagy using 3-methyladenine and enhancing its biogenesis using pioglitazone reversed NSP1-mediated inhibition of ISG expression. Pioglitazone is a selective PPARγ agonist and is used clinically for the treatment of diabetes (62), suggesting that it may become a candidate drug for the clinical treatment of ALSV infection.

Recently, there has been a growing recognition of the roles of STATs in mitochondrial function. STAT3 localizes to mitochondria and is implicated in the functioning of OXPHOS complexes to optimize mitochondrial bioenergetics (63, 64). STAT2 has also been identified within mitochondria (65). Upon viral infection, STAT2 translocates to mitochondria, where it is targeted for degradation by the viral degradasome formed by viral proteins and MAVS (66). Additionally, patients harboring clinically asymptomatic STAT2 mutations were reported to exhibit severe neurological deterioration following viral infection, which was attributed to STAT2 deficiency and which impeded mitochondrial fission (39, 40). Furthermore, STAT2 induced by LPS has been found to increase mitochondrial mass by enhancing DRP1 phosphorylation at Ser616. Thus, in turn, STAT2 promotes mitochondrial fission and biogenesis, which are essential for the pro-inflammatory differentiation of macrophages (31). Here, we have confirmed that ALSV relies on STAT2 to reduce mitochondrial mass. This discovery enhances our understanding of the intricate relationship between STAT2, mitochondrial function, and viral infection. However, the precise mechanism by which NSP1 causes the reduction of mitochondrial mass remains to be explored. It is worth noting that STAT2 could interact with OPA1 but not with DRP1 or FIS1 (Fig. 7H). Moreover, NSP1 does not promote the migration of STAT2 to mitochondria (Fig. 7I). Further investigations are needed to fully elucidate the specific mechanism involved.

While our study primarily investigated how ALSV evades the host’s IFN-I response to enhance its replication, it is important to note that Vero cells do not support sustained viral replication. This observation suggests that factors beyond IFN-I signaling also contribute to the inhibition of viral replication in mammalian-derived cells. These factors may include, but are not limited to, virus entry receptors and cellular restriction factors that interfere with viral entry, replication, or assembly, all of which play critical roles during viral infection and replication (67). Furthermore, the differing replication capabilities of ALSV in mammalian versus tick-derived cells may be influenced by their distinct growth cycles: approximately 10 days for tick-derived cells compared to 0.9–2 days for mammalian-derived cells (68, 69).

ALSV has been shown to infect humans, resulting in viremia and varying degrees of clinical symptoms; however, all infected individuals ultimately recovered completely, indicating that ALSV is currently self-limiting (4). This observation aligns with relatively weak replication capability of ALSV in human cells. In cases where patients have severe clinical symptoms, these may be the result of a combination of viral infection and pre-existing underlying health conditions. Nonetheless, it remains a concern whether the virus could potentially mutate and evolve, thereby breaching this barrier and leading to a pandemic.

In summary, ALSV is sensitive to IFN-β and possesses the ability to antagonize the IFN-I response. At the mechanistic level, ALSV NSP1 can selectively bind to and degrade human STAT2 through an autophagy pathway. This degradation directly hampers the expression of ISGs and leads to a reduction in mitochondrial mass by perturbing mitochondrial dynamics to induce mitophagy and suppressing its biogenesis, ultimately resulting in the dampening of the antiviral response. In addition, promoting mitochondrial mass by inhibition of mitophagy using 3-methyladenine and enhancing its biogenesis using pioglitazone can reverse NSP1-mediated antagonism of the interferon response, suggesting a potential intervention strategy for ALSV infection. Our findings elucidate the intricate regulatory cross talk between ALSV and the host’s innate immune response, providing valuable insights into the pathogenesis of, and intervention strategy against, this emerging segmented flavivirus.

## MATERIALS AND METHODS

### Cells

Human embryonic kidney (HEK293T) cells (ATCC CRL-3216), human hepatoblastoma (HepG2) cells (ATCC HB-8065), human lung epithelial (A549) cells (ATCC CCL-185), and mouse leukemic monocyte/macrophage (Raw264.7) cells (ATCC TIB-71) were grown in Dulbecco’s modified Eagle’s medium (DMEM, high glucose) (Sigma-Aldrich, cat# R8758-500ML), supplemented with 10% fetal bovine serum (FBS) (BBI, cat# E600001) and antibiotics (100 units/mL penicillin and 100 µg/mL streptomycin, PS) (Sangon, cat# B540732). African green monkey kidney (Vero) cells (*Chlorocebus sabaeus*, JCRB0111) were maintained in DMEM (high glucose) supplemented with 10% FBS and PS. STAT2^-/-^ cells, which are STAT2 knockout in HEK293 cells, were maintained in DMEM containing 10% FBS, puromycin (2 µg/mL; YEASEN, cat# 60210ES25), and PS. All of the aforementioned cells were cultured at 37°C in an atmosphere of 5% CO_2_ and routinely tested negative for mycoplasma contamination. To induce an IFN-I response, the target cells were treated with 10 ng/mL recombinant human IFN-β (PeproTech, Cat# 300-02BC) for the specified durations. The *Ixodes ricinus* embryo-derived tick cell line IRE/CTVM19 (70) was cultured in L15 (Leibovitz) medium supplemented with 20% heat-inactivated FBS (Sigma, cat# F8318), 10% tryptose phosphate broth, 2 mM L-glutamine and PS, and was maintained in sealed containers in ambient air at 30°C.

### Plasmid construction

The cDNAs corresponding to the viral protein sequences of ALSV were synthesized by Sangon and subcloned into the Xba I site (YEASEN, Cat# 15033ES76) of 3 × Flag-VR1012 expression vector (BioVector NTCC, Cat# VR1012) using the Hieff Clone Plus One Step Cloning Kit (YEASEN, Cat# 10911) (18). To construct the expression plasmid for ALSV NSP1 MTase (1-280 aa) and RdRp (281-419 aa) domains, the cDNAs were amplified from the Flag-NSP1 template and subsequently cloned into 3 × Flag-VR1012 using Hieff Clone Plus One Step Cloning Kit. For the construction of the plasmid for NSP1 lentiviral transduction, the cDNA of NSP1 was amplified from the Flag-NSP1 template and cloned into the lentiviral vector pWPI (Addgene, Cat# 12254). A plasmid expressing the human STAT2 mutant (HA-STAT2-F175A/R176A) was generated through site-directed mutagenesis PCR using HA-STAT2-Myc (MIAOLING BIOLOGY, Cat# P4105) as a template. To clone mouse STAT2, total RNA was isolated from Raw264.7 cells using the EasyPure RNA Kit (TransGen, cat# ER101). The synthesized cDNA was PCR amplified using STAT2-specific primers and cloned into the 3 × HA-VR1012 expression vector. Nucleotide sequences were determined by a DNA sequencing service provided by Sangon. Detailed primer sequences are available upon request. The pSTAT1-Luc and pSTAT2-Luc expression plasmids were kindly provided by Dr. Guangyun Tan. All constructed plasmids were transformed into Stbl3 (Thermo Fisher Scientific, Cat# C737303) or Trans5α (TransGen, cat# CD201-01) chemically competent cells, followed by selection on antibiotic-containing Luria-Bertani (LB) agar plates.

### ALSV infection

ALSV was isolated from a patient who had been bitten by a tick in Northeast China (4). Working virus stocks of ALSV were prepared from IRE/CTVM19 cells. In brief, 1 mL of the seed virus strain H3 derived from infected Vero cells was inoculated into IRE/CTVM19 cells. At 1 hpi, an additional 2 mL of fresh L15 complete culture medium was added to the cells. At 7 days post-infection, the culture medium was harvested by centrifugation at 15,000 × *g* for 30 minutes at 4°C, and the supernatant was collected to create the working virus stock. It is impossible to detect the virus titer by plaque assay or TCID_50_ as ALSV does not produce cytopathic effect (CPE) in mammalian cells. Instead, we detected the ability of the virus to infect cells through flow cytometry using the anti-dsRNA antibody (Fig. S1C). We obtained the titer of the ALSV stock as 2.56 × 10^6^ transducing units/mL by the formula TU/mL = (*F* × *Ci* / *V*) × *D*, where *F* is the positive cell rate, *Ci* is the total number of cells at the time of transduction, *V* is the volume of the inoculated virus, and *D* is the viral dilution factor. In addition, the vRNA copies of the ALSV stock were 4.86 × 10^8^ copies/μL detected by Taqman-qPCR. All experiments involving infectious ALSV were conducted in strict compliance with biosafety level 2 conditions. Manipulations involving both inactivated and non-inactivated ALSV followed the guidelines and regulations set forth by the Chinese authorities regarding dual-use pathogens.

One day before infection, HEK293T cells, STAT2^-/-^, A549, HepG2, and Raw264.7 cells were infected with ALSV virus stock at the indicated multiplicity of infection (MOI) or MOI 2.5, and were incubated at 37°C in serum-free DMEM (high glucose) for 2 hours. The infected cells were subsequently washed twice with PBS and cultured with fresh DMEM (high glucose) containing 2% FBS and 1% PS. For viral RNA quantification, 150 µL of culture supernatant was harvested at the specific timepoints and subjected to Taqman-qPCR using ALSV segment 2 (*S2*)-specific primers: 5′-GCTTGTGGTCATCATTATG-3′ (forward), 5′-CTCTGCCACATACTGATG-3′ (reverse), and 5′-CTCTCGTCAGCCATACCACCA-3′ (probe primer). Briefly, viral RNA was extracted from mock- or ALSV-infected cell-culture supernatants using a viral RNA extraction kit (TIANGEN, Cat# DP315), and was transcribed into cDNA using the cDNA Synthesis SuperMix (TransGen, cat# AT341). TaqMan-qPCR was performed using the Premix Ex Taq (Probe qPCR) Kit (TaKaRa, cat# RR390L) in an StepOne Plus Real-Time PCR system (Applied Biosystem, USA) with the following cycling conditions: 95°C for 30 seconds, followed by 40 cycles of denaturation at 95°C for 5 seconds and annealing/extension at 60°C for 30 seconds. The quantitative conversion of virus copy numbers from the Ct value was determined using the formula of (copies)/μL = 10^([value of Ct – 9.01]/–2.895), which was generated by constructing a standard curve with DNA derived from an ALSV S2-expressing plasmid stock with a known plasmid concentration. All mock-infected samples exhibited non-singular melting curves, indicating no non-specific amplification, and the values for these samples were set to zero.

Following treatment, the mock- or ALSV-infected cells were collected and used for SYBR-qPCR to quantify the RNA level of the viral S2 gene (see “RT-qPCR” section below). Additionally, immunofluorescence analysis was performed on the mock- or ALSV-infected cells to detect viral dsRNA levels.

### Infectivity assay

To determine the infectivity of the virus secreted by mammalian cells infected with ALSV, a virus infectivity assay was conducted. Briefly, Vero cells were infected with ALSV at an MOI of 4. After 2–3 days, the collected supernatants, designated as passage 1, were subcultured without dilution up to passage 3. These supernatants were then used to infect IRE/CVM19 tick cells. At 6 days post-infection, the cells were stained with an anti-VP2 antibody and counterstained with DAPI for nuclear visualization. Finally, the cells were observed under a fluorescence microscope.

### Quantitative real-time PCR (RT-qPCR)

Quantitative real-time PCR (qPCR) was performed using the primers listed in the Supplemental Key Resources Table, following the established protocols (71). In brief, after the specified treatment as indicated in the figure legends, cells were rinsed twice with PBS and then lysed using a lysis buffer. Total RNA was extracted according to the manufacturer’s instructions using the EasyPure RNA Kit. The extracted RNA was subsequently reverse transcribed into cDNA using the cDNA Synthesis SuperMix (TransGen, cat# AT341). For the qPCR step, Fast SYBR Green Master Mix (Roche, cat# 4913850001) was used, and the reactions were run on a Step-One Plus real-time PCR system. The PCR program started with activation at 95°C for 5 minutes, followed by 40 cycles of denaturation at 95°C for 10 seconds and annealing/extension at 60°C for 30 seconds. The glyceraldehyde 3-phosphate dehydrogenase (GAPDH) gene was employed as the reference gene for normalization. For experiments involving the induction of ISGs measured via qPCR, the target cells were treated with 10 ng/mL IFN-β for the specific durations.

### Dual-luciferase reporter assay

For the ALSV infection experiment measured via dual-luciferase reporter assay (Fig. 2G), HEK293T cells were seeded in 24-well plates at a density of 2 × 10^5^ per well. They were co-transfected with 250 ng of the IFN-β inducible firefly luciferase reporter plasmid (ISRE-luc) and 50 ng of pGL4.74, which constitutively expresses Renilla luciferase. Transfection was performed using polyethylenimine (PEI) (YEASEN, Cat# 40816ES02) following the manufacturer’s instructions. At 24 hpt, cells were infected with ALSV and subsequently treated with IFN-β (10 ng/mL) for either 24 or 48 hours. Then, cells were harvested, and the luciferase activity was measured using a dual-luciferase reporter assay (Promega, cat# E1910). For experiments involving the transfection of viral proteins (Fig. 3B and 4F; Fig. S1A), HEK293T cells cultured in 24-well plates were co-transfected with 500 ng of the indicated plasmids expressing viral proteins, along with 250 ng of ISRE-luc and 50 ng of pGL4.74, all using PEI. At 24 hpt, cells were treated with IFN-β for 12 hours and subsequently analyzed for reporter activity. Average firefly luciferase values were normalized to average Renilla luciferase values. Mock or empty vector-treated samples without IFN-β treatment were set to 1, and each sample’s luciferase activity was standardized to this value.

### Immunoblotting

For all experiments, HEK293T cells were used, except for Figure 7B, in which RAW264.7 cells were utilized. HEK293T or STAT2^-/-^ cells were either infected with ALSV or transfected with Flag-tagged NSP1, with or without the indicated plasmids. At 2 hpi or 24 hpt, cells were either mock treated or treated with 10 ng/mL IFN-β for the specific duration and subsequently analyzed by immunoblotting.

For the STAT2 degradation inhibitor experiment, HEK293T cells transfected with Flag-tagged NSP1 or vector plasmid were treated with the following compounds for 6 hours: 10 µM of MG132 (Merck, Cat# 474787), 50 µM of chloroquine (CQ, MCE, Cat# HY-17589A), 10 mM of 3-methyladenine (3-MA, Selleck, Cat# S2767), or 50 µM of Z-VAD-FMK (Selleck, Cat# S7023). In most cases, cells were lysed for 30 minutes using a lysis buffer (containing 50 mM Tris-HCl, pH 8.0, 150 mM NaCl, 1% NP-40) supplemented with a protease inhibitor cocktail (Selleck, cat# B14002). To detect phosphorylated proteins, cells were lysed using the same lysis buffer, but with Halt Protease and Phosphatase Inhibitor Single-Use Cocktail (Thermo Fisher Scientific, cat# 78442). The cell lysates were then centrifuged at 12,000 × *g* for 10 minutes at 4°C, and the protein concentration was determined using a Pierce BCA Protein Assay Kit (Thermo Fisher Scientific, cat# 23225). The lysates were subsequently reduced with 1× Protein Loading buffer (TransGen, cat# DL101-02) for 5 minutes at 95°C. For electrophoresis, 20–30 μg of protein for each sample was separated by 8%–15% sodium dodecyl sulfate-polyacrylamide gel electrophoresis (SDS-PAGE) and was transferred onto PVDF membranes (Millipore, cat# IPVH00010) using Trans-Blot Systems (Bio-Rad). Following transfer, the membranes were blocked with 2% bovine serum albumin (BSA) (VETEC, cat# V900933) in PBS containing 0.2% Tween-20 (PBST) and then incubated with appropriate primary antibody at 4°C overnight. After washing, the membranes were incubated with horseradish peroxidase (HRP)-conjugated secondary antibodies, and antibody–antigen complexes were visualized using a chemiluminescence (ECL) substrate (Millipore, cat# WBKlS0100). The grayscale analysis of the bands was performed with ImageJ software.

### Co-immunoprecipitation (Co-IP) assays

For the Co-IP assays, HEK293T cells were transfected with the specified plasmids. At 24 hpt, cells were treated with or without CQ for 12 hours prior to the Co-IP procedure. Lysates were collected and centrifuged at 12,000 × *g* for 10 minutes at 4°C to obtain a whole-cell extract. The remaining lysate was then incubated with either anti-Flag M2 Affinity Gel (Sigma-Aldrich, cat# A2220) or anti-HA Affinity Gel (Millipore, cat# E6779) at 4°C overnight to facilitate the immunoprecipitation process. The binding beads were washed several times with lysis buffer and subsequently denatured in 1× protein loading buffer for 10 minutes. Finally, the proteins within immunocomplexes and whole-cell extracts were analyzed using immunoblotting with the appropriate antibodies.

### Cell fractionation extraction

The nuclear and cytoplasmic protein extraction process was performed as previously described (72). Briefly, HEK293T cells transfected with Flag-NSP1 and HA-STAT2 plasmids were treated either with or without IFN-β for 30 minutes. Subsequently, they underwent fractionation extraction using the Nuclear and Cytoplasmic Protein Extraction Kit (Beyotime, cat# P0027) according to the manufacturer’s instructions. The purified nuclear and cytoplasmic proteins, along with total lysates, were then subjected to immunoblotting assays with specific antibodies against Flag, HA, GAPDH, and Histone. For mitochondrial isolation, HEK293T cells were transfected with either Flag-tagged NSP1 or vector plasmid. At 48 hpt, the cells underwent mitochondrial extraction using the Cell Mitochondria Isolation Kit (Beyotime, cat# C3601). The isolated mitochondria were subsequently analyzed using immunoblotting.

### Immunofluorescence assays

For viral infection experiments, target cells were seeded onto coverslips in 24-well culture plates at a density of 1 × 10^5^ per well and then infected with ALSV. At 2 hpi, the virus was removed, and the cells were replenished with DMEM medium containing 10 ng/mL IFN-β. After 24 hours, cells were fixed with 4% paraformaldehyde (PFA, Biotopped, cat# Top0382) for 30 minutes. Subsequently, the cells were permeabilized with 0.1% Triton X-100 (YEASEN, cat# 20107ES76) for 15 minutes and blocked with 1% BSA for 2 hours. Following blocking, the cells were stained with anti-dsRNA antibody (SCICONS, Cat# 10010200) and an Alexa Fluor 488-conjugated anti-mouse secondary antibody (Proteintech, Cat# SA00013-1). Finally, nucleic acids within the cells were counterstained with 4′,6-diamidino-2-phenylindole (DAPI) (YEASEN, cat# 40728ES03). To detect STAT2 nuclear transfer, HepG2 cells were transfected with NSP1 or vector plasmid. At 24 hpt, cells were treated with or without IFN-β for 30 minutes, then they were fixed, permeabilized, blocked, and incubated with the anti-Flag and STAT2 antibodies. For the observation of GFP-LC3 dot formation, HepG2 cells expressing GFP-LC3B were either transfected with Flag-NSP1 for 48 hours or treated with CQ for 16 hours, followed by immunostaining. Cells were visualized using confocal microscopy (FV3000, OLYMPUS), and the acquired images were analyzed with ImageJ software.

### Assessment of mitochondrial mass

One day before infection or transfection, HEK293T, STAT2^-/-^, A549, and HepG2 cells were seeded into 24-well plates. The cells were then either infected with ALSV or transfected with the plasmid pWPI-NSP1, which has a GFP tag. After 48 hours post-treatment, the cells were incubated with MTR at a final concentration of 100 nM for 30 minutes at 37°C. Following incubation, the cells were washed three times with PBS for 5 minutes each and then analyzed by flow cytometry (LSRFortessa, BD Biosciences, Maryland, USA). The MFI of MTR was analyzed using the FlowJo software. Additionally, the mitochondrial mass was quantified by immunofluorescence assay. Briefly, the cells transfected with the Flag-NSP1 plasmid were fixed and immunostained with anti-Flag and COXIV antibodies after MTR treatment, and the cells infected with ALSV were immunostained with anti-dsRNA and STAT2 antibodies after MTR incubation. Fluorescence images were captured using a confocal microscope. The MFI for infected cells and the total fluorescence intensity (TFI) per cells for transfected cells were quantified using ImageJ software.

### Lentiviral packaging and transduction

The STAT2 knockout plasmid, pLenti-STAT2-sgRNA (Beyotime, cat# L00231), which simultaneously expresses Cas9, sgRNA for the target gene, and puromycin resistance, was obtained from Beyotime Biotechnology (Shanghai, China). Lentiviral particles were generated by transfecting 8.63 µg of the pLenti-STAT2-sgRNA plasmid, along with 3.1 µg of pMD2.G (Addgene, Cat# 12259) and 5.5 µg of psPAX2 (Addgene, Cat# 12260), into HEK293T cells (6 × 10^6^ cells) cultured in a 10-cm dish. The transfection was carried out using PEI transfection reagent according to the manufacturer’s protocol. At 48 and 72 hpt, the culture supernatant was harvested and centrifuged, and cell debris was removed by passing it through a 0.45-µm pore size filter (Merck, Cat# SLGVR33RB). Subsequently, lentiviral particles were inoculated into target cells and incubated at 37°C for 48 hours. The cells were then selected using culture medium containing 2 µg/mL puromycin (YEASEN, Cat# 60210ES25) for a period of 2 weeks. After expansion, the expression level of STAT2 was verified through immunoblotting. Simultaneously, the LentiCRISPRv2 plasmid (Addgene, Cat# 52961) was used as an empty vector to establish the control cell line.

To generate lentiviral particles overexpressing ALSV NSP1, HEK293T cells were co-transfected with pWPI-NSP1 or empty pWPI along with pMD2.G and psPAX2 in a ratio of 2.8:1:1.8 using PEI. Viral supernatant was collected at 48 and 72 hpt. The relative infectivity was determined by flow cytometry, measuring the percentage of GFP-positive cells in HEK293T cells infected with serial dilutions of the lentiviral particles for 48 hours.

### Quantification and statistical analysis

Data analyses were conducted using Prism 9.0.2 (GraphPad Software). The data are presented as the mean ± standard deviation (SD), and *n* represents the number of technical replicates. Statistically significant differences were determined by one- or two-way analysis of variance (ANOVA) with multiple comparisons correction. Significance is indicated by asterisks, as follows: *P < 0.05, **P < 0.01, ***P < 0.001, ****P < 0.0001, and ns denotes non-significant differences.

## Data Availability

The data supporting the conclusions of this article are included within the article and its supplemental material or are available from the authors upon reasonable request.

## SUPPLEMENTAL MATERIAL

Supplemental material jvi.01301-24-s0001.docx Figures S1 to S5; table of key resources.

ASM does not own the copyrights to Supplemental Material that may be linked to, or accessed through, an article. The authors have granted ASM a non-exclusive, world-wide license to publish the Supplemental Material files. Please contact the corresponding author directly for reuse.
