# Supplementary material for: The segmented flavivirus Alongshan virus reduces mitochondrial mass by degrading STAT2 to suppress the innate immune response
Source: J Virol. 2024 Dec 10;99(1):e01301-24. doi: 10.1128/jvi.01301-24 (PMC11784234; doi:10.1128/jvi.01301-24)
Supplement: Supplemental material — Figures S1 to S5; table of key resources. [file jvi.01301-24-s0001.docx]

Supplementary Materials for

**The segmented flavivirus Alongshan virus reduces mitochondrial mass by degrading STAT2 to suppress the innate immune response**


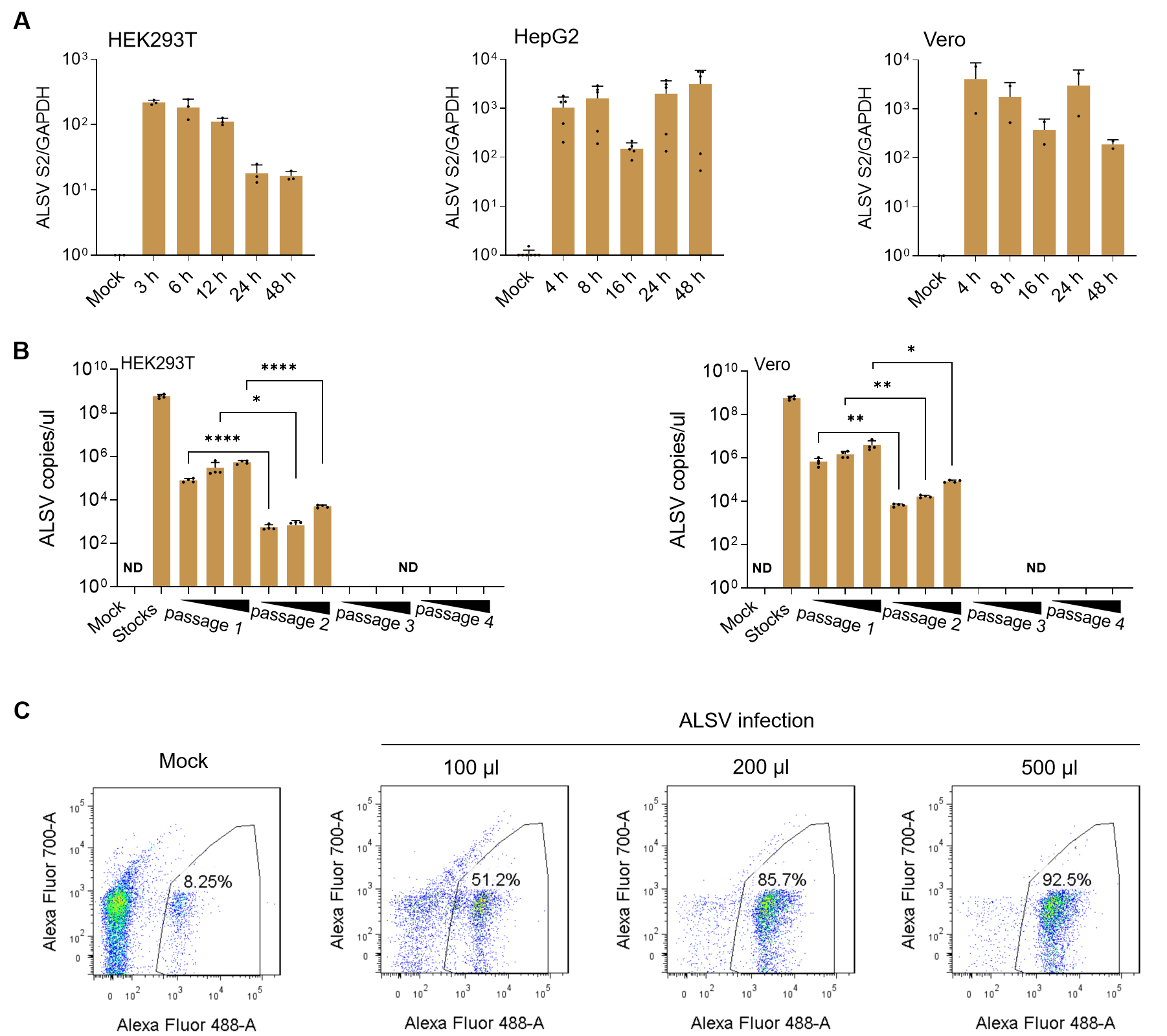


**FIG S1** The infection and replication capability of Alongshan virus (ALSV) in mammalian cells. (A) The ALSV infectivity assay by flow cytometry using the anti-dsRNA antibody. Vero cells cultured in 12-well plates were infected with escalating doses of ALSV. At 48 hours post-infection (hpi), cells were analyzed by flow cytometry using an anti-dsRNA antibody, and an Alexa Fluor® 488 conjugated anti-mouse IgG was used as a secondary antibody. (B) HEK293T, HepG2 and Vero cells were infected with ALSV, the mRNA levels of viral segment 2 (*S2*) relative to *GAPDH* control in cells were measured by qPCR at the indicated time points. (C) HEK293T and Vero cells were infected with the escalating doses of ALSV (MOI 4, 2, 1). After 2-3 days, the collected culture supernatants were used as passage 1, which were then subcultured without dilution to passage 4. The ability of the virus to replicate was assessed in supernatants by TaqMan-qPCR. Statistical analysis was performed using one-way ANOVA with multiple comparison correction (**P* < 0.05, ***P* < 0.01, and *****P* < 0.0001).


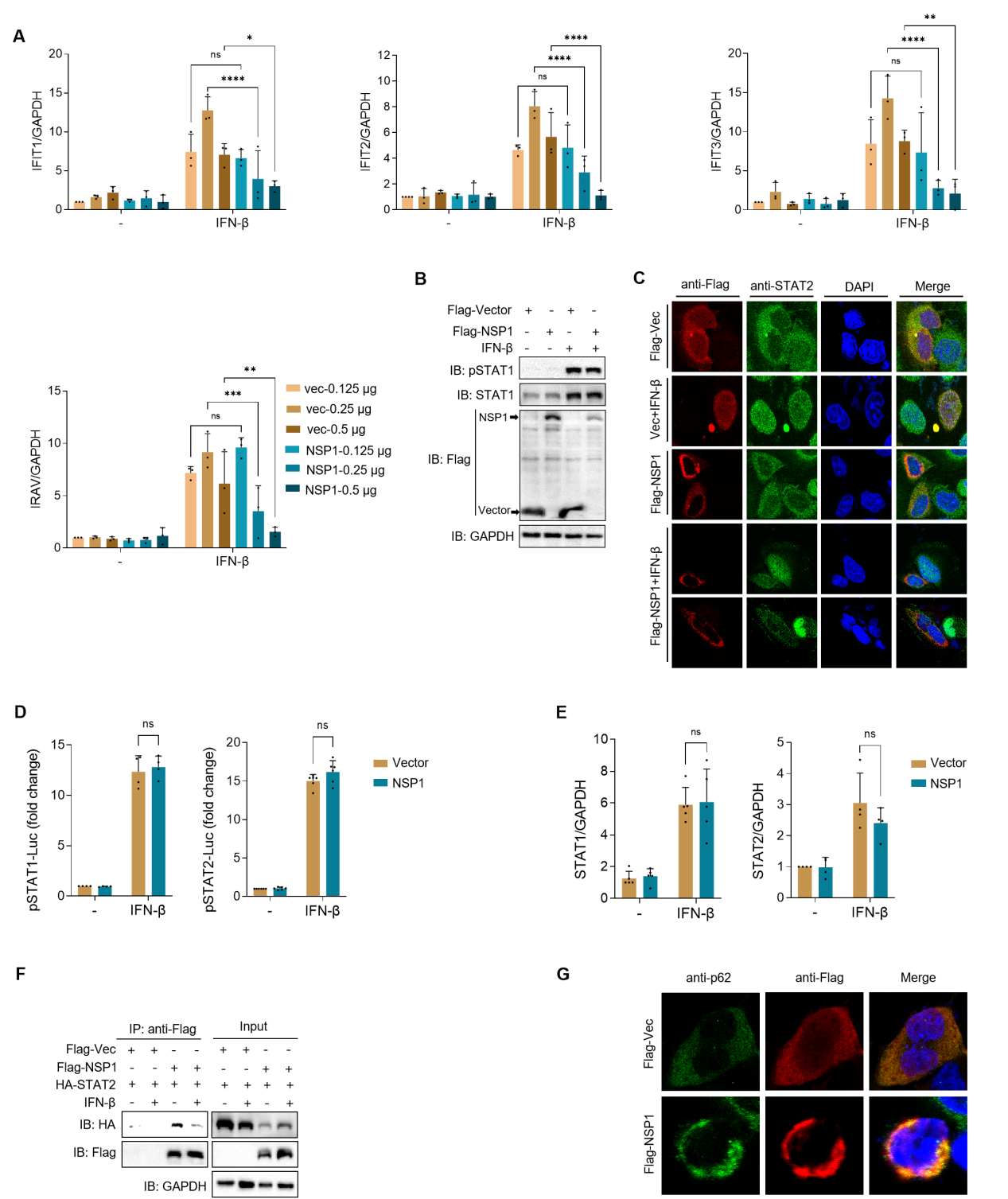


**FIG S2** Alongshan virus **(**ALSV) NSP1 protein suppresses IFN-β-induced ISG expression. (A) HEK293T cells cultured in 24-well plates were transfected with escalating doses of NSP1 or vector plasmid. After 24 h, cells were treated with or without IFN-β for 12 h, the mRNA expression of *IFIT1*, *IFIT2*, *IFIT3*, and *IRAV* was examined using qPCR. (B) HEK293T cells were transfected with Flag-tagged NSP1 or vector plasmid. At 24 hours post-transfection (hpt), cells were treated with or without IFN-β for 12 h, cell lysates were analyzed by immunoblotting with the indicated antibodies, with GAPDH as a loading control. (C) HepG2 cells were transfected with NSP1 or vector plasmid. At 24 hpt, cells were treated with or without IFN-β for 30 min, and cells were subjected to immunofluorescence with anti- STAT2 and Flag antibodies. Nuclei were counterstained with DAPI. (D) HEK293T cells were co-transfected with STAT1 or STAT2 promoter and NSP1 plasmids. At 24 hpt, cells were treated with or without IFN-β for 12 h, the cells were harvested and luciferase activity was measured. (E) HEK293T cells were transfected with NSP1 or vector plasmid. At 24 hpt, cells were treated with or without IFN-β for 12 h, the mRNA expression of *STAT1* and *STAT2* was examined using qPCR. (F) HEK293T cells were transfected with the indicated plasmids. At 24 hpt, cells were treated with or without IFN-β for 12 h, the anti-Flag immunoprecipitates were analyzed by immunoblotting with HA and Flag antibodies. (G) Immunofluorescence of Flag-NSP1 or vector-transfected HepG2 cells stained for p62 and Flag. Statistical analysis was conducted using two-way ANOVA with multiple comparison correction (**P* < 0.05, ***P* < 0.01, ****P* < 0.001, and *****P* < 0.0001).


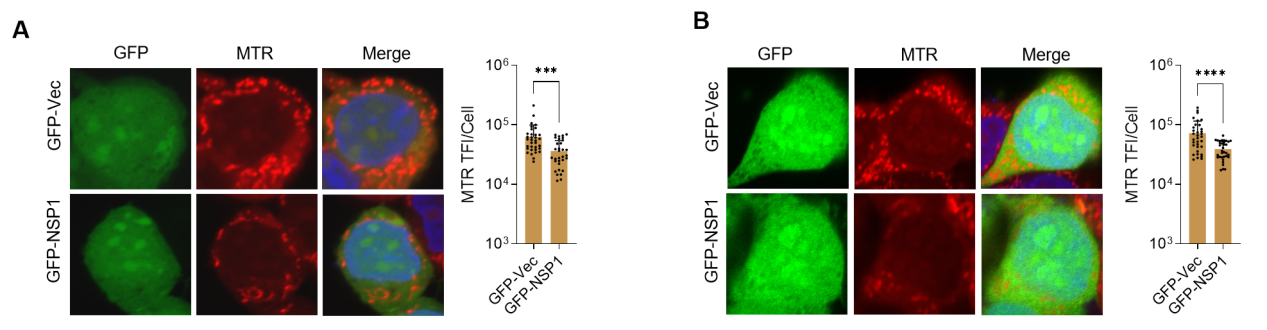


**FIG S3** Alongshan virus **(**ALSV) NSP1 reduces mitochondrial mass. (A and B) HEK293T (A) and A549 (B) cells were transfected with NSP1 plasmid with an unfused GFP tag. At 48 hpt, cells were stained with MitoTracker-Red (MTR) and examined by laser scanning confocal microscopy. The total fluorescence intensity (TFI) of MTR per cell was analyzed (n≥20 cells). Statistical analysis was conducted using one-way ANOVA with multiple comparison correction (****P* < 0.001 and *****P* < 0.0001).


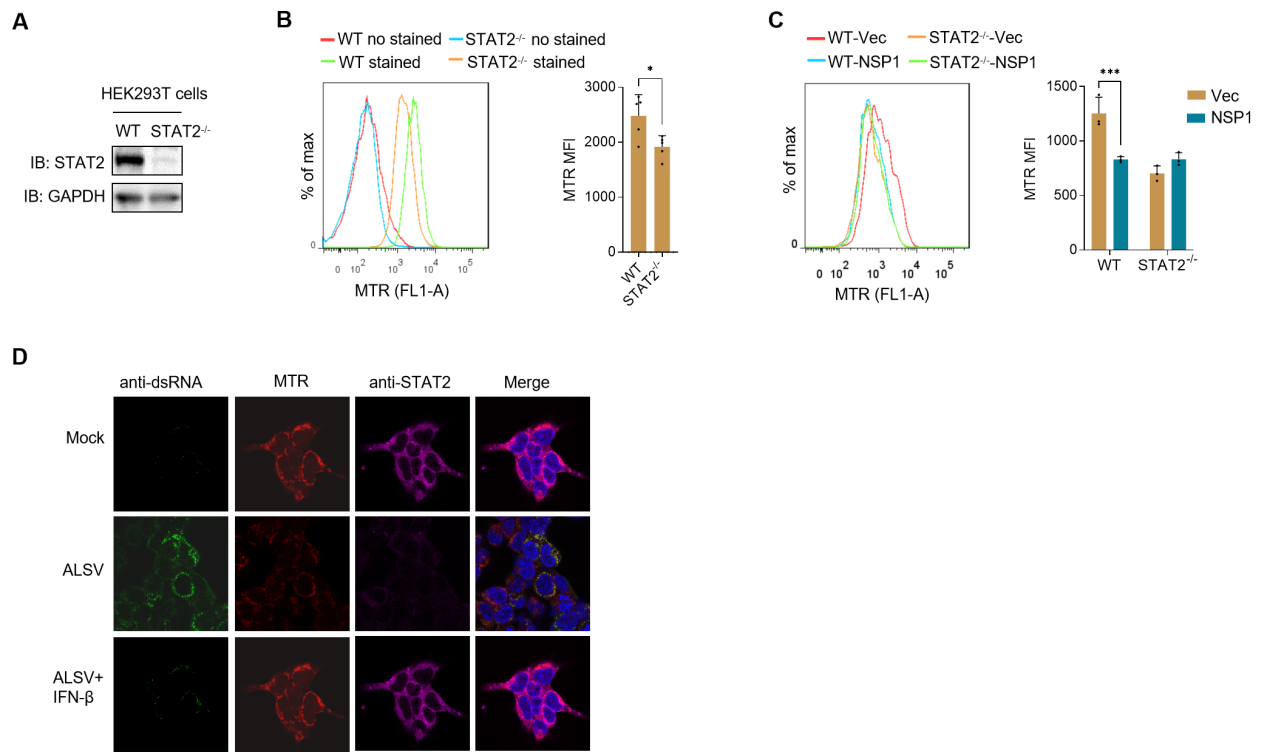


**FIG S4** Alongshan virus **(**ALSV) NSP1 reduces mitochondrial mass in a STAT2-dependent manner. (A) HEK293T wild-type (WT) or STAT2 knockout (STAT2^-/-^) cells were analyzed by immunoblotting with anti-STAT2 antibody. (B) WT or STAT2^-/-^ cells were stained with MitoTracker-Red (MTR) and analyzed by flow cytometry. The mean fluorescence intensity (MFI) of MTR was measured (n=5). (C) WT or STAT2^-/-^ cells were infected with lentiviral particles expressing GFP-tagged NSP1 or vector and incubated for 48 h. Cells were stained with MTR and analyzed by flow cytometry. The MTR MFI was measured in GFP^+^ cells (n=3). (D) A549 cells were infected with ALSV. At 2 hpi, cells were treated with or without IFN-β for 24 h. Cells were stained with anti- dsRNA and STAT2 antibodies along with MTR. Statistical analysis was performed using one or two-way ANOVA with multiple comparison correction (**P* < 0.05 and ****P* < 0.001).


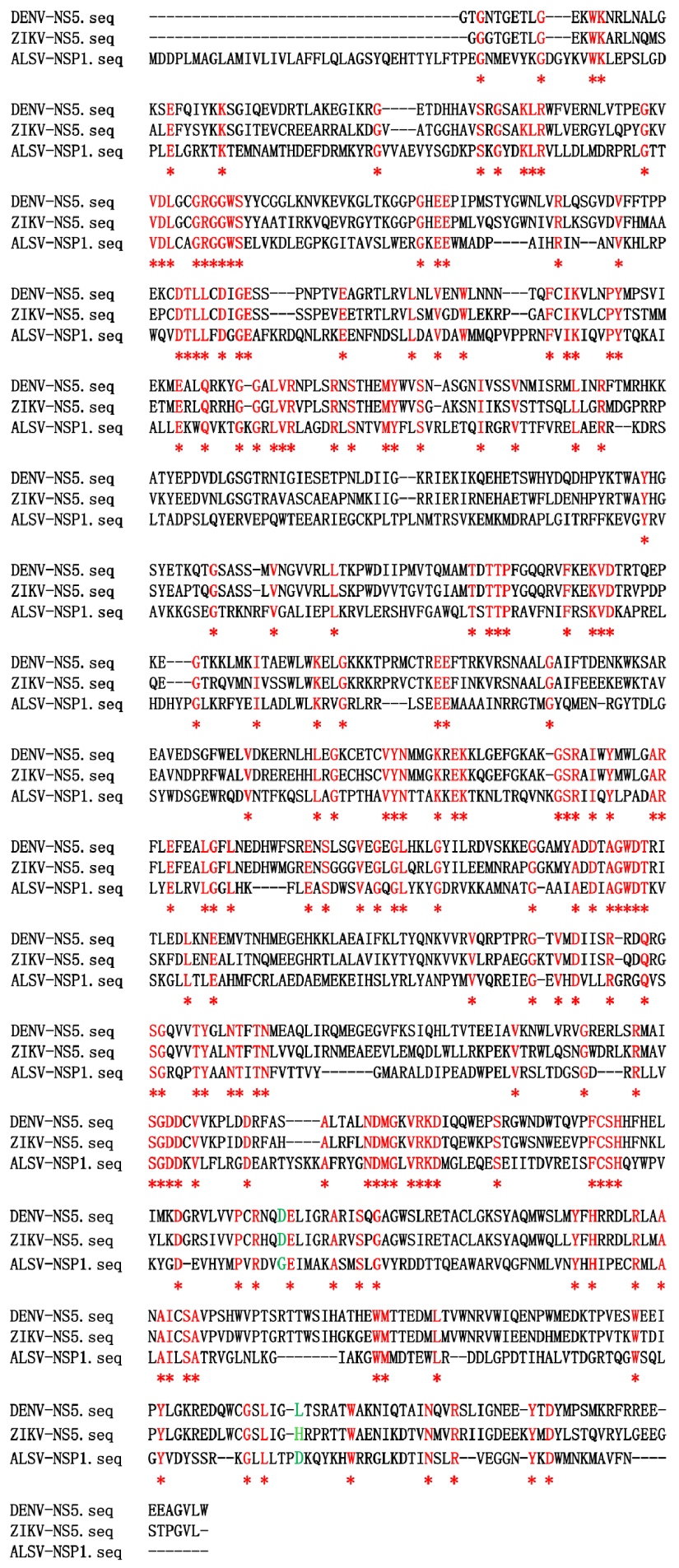


**FIG S5** Sequence alignment of Alongshan virus (ALSV) NSP1 protein with DENV and ZIKV NS5 proteins. A sequence alignment of the ALSV NSP1 with the NS5 proteins of DENV and ZIKV. Identical residues among these proteins are marked by red asterisks, highlighting the regions of similarity. The hSTAT2-binding sites identified in the NS5 proteins of ALSV, ZIKV and DENV are colored in green**.**

**KEY RESOURCES TABLE**

| REAGENT or RESOURCE | SOURCE | IDENTIFIER |
| --- | --- | --- |
| Antibodies |  |  |
| Rabbit polyclonal anti-GST tag | Proteintech | Cat#10000-0-AP; RRID:AB_11042316 |
| Mouse monoclonal anti-FLAG tag | Proteintech | Cat#66008-3-Ig; RRID:AB_2749837 |
| Rabbit polyclonal anti-FLAG tag | Proteintech | Cat#20543-1-AP; RRID:AB_11232216 |
| Rabbit polyclonal anti-HA tag | Proteintech | Cat#51064-2-AP; RRID:AB_11042321 |
| Mouse monoclonal anti-HA tag | Proteintech | Cat#66006-2-Ig; RRID:AB_2881490 |
| Rabbit polyclonal anti-IFIT3 | Proteintech | Cat#15201-1-AP; RRID:AB_2248738 |
| Rabbit polyclonal anti-IRAV | Proteintech | Cat#27865-1-AP; RRID:AB_2880997 |
| Mouse monoclonal anti-GAPDH | Proteintech | Cat#60004-1-Ig; RRID:AB_2107436 |
| Rabbit polyclonal anti-GAPDH | Proteintech | Cat#10494-1-AP; RRID:AB_2263076 |
| CoraLite594 – conjugated Goat Anti-Rabbit IgG(H+L) | Proteintech | Cat#SA00013-4; RRID:AB_2810984 |
| CoraLite594 – conjugated Goat Anti-Mouse IgG(H+L) | Proteintech | Cat# SA00013-3, RRID:AB_2797133 |
| CoraLite488-conjugated Goat Anti-Rabbit IgG(H+L) | Proteintech | Cat# SA00013-2, RRID:AB_2797132 |
| CoraLite488-conjugated Goat Anti-Mouse IgG(H+L) | Proteintech | Cat# SA00013-1, RRID:AB_2810983 |
| IFIT1 (D2X9Z) Rabbit mAb | Cell Signaling Technology | Cat# 14769, RRID:AB_2783869 |
| Rabbit polyclonal anti-STAT2 | Abcam | Cat# ab32367, RRID:AB_778098 |
| Anti-phospho-STAT2 (Tyr689) Antibody | Millipore | Cat# 07-224, RRID:AB_2198439 |
| Monoclonal anti-dsRNA Antibody (J2) | SCICONS | Cat# 10010200, RRID:AB_2651015 |
| Rabbit polyclonal anti-STAT1 | Proteintech | Cat# 10144-2-AP, RRID:AB_2286875 |
| Phospho-Stat1 (Tyr701) (58D6) Rabbit mAb | Cell Signaling Technology | Cat# 9167, RRID:AB_561284 |
| LC3B (D11) XP® Rabbit mAb | Cell Signaling Technology | Cat# 3868, RRID:AB_2137707 |
| Rabbit polyclonal anti-p62 | Proteintech | Cat# 18420-1-AP, RRID:AB_10694431 |
| Rabbit polyclonal anti-GFP | Proteintech | Cat# 50430-2-AP, RRID:AB_11042881 |
| Rabbit polyclonal anti-COXIV | Proteintech | Cat# 11242-1-AP, RRID:AB_2085278 |
| Rabbit polyclonal anti-TOM20 | Proteintech | Cat# 11802-1-AP, RRID:AB_2207530 |
| Rabbit polyclonal anti-Tim23 | Proteintech | Cat# 11123-1-AP, RRID:AB_615045 |
| Rabbit polyclonal anti-calnexin | Proteintech | Cat# 10427-2-AP, RRID:AB_2069033 |
| Rabbit monoclonal anti-DPR1 | ABclonal | Cat# A21968, RRID: AB_3073826 |
| Phospho-DRP1-S616 Rabbit mAb | ABclonal | Cat# AP1353, RRID: AB_3073823 |
| Phospho-DRP1 (Ser637) Rabbit pAb | Zen Bio | Cat# 310300, RRID:AB_3073818 |
| Rabbit polyclonal anti-FIS1 | Proteintech | Cat# 10956-1-AP, RRID:AB_2102532 |
| Rabbit polyclonal anti-MFN1 | Proteintech | Cat# 13798-1-AP, RRID:AB_2266318 |
| Rabbit polyclonal anti-MFN2 | Proteintech | Cat# 12186-1-AP, RRID:AB_2266320 |
| Rabbit polyclonal anti-OPA1 | Proteintech | Cat# 27733-1-AP, RRID:AB_2810292 |
| Mouse monoclonal anti-PGC1α | Proteintech | Cat# 66369-1-Ig, RRID:AB_2828002 |
| Rabbit polyclonal anti-NRF1 | Proteintech | Cat# 12482-1-AP, RRID:AB_2282876 |
| Rabbit polyclonal anti-TFAM | Proteintech | Cat# 22586-1-AP, RRID:AB_11182588 |
| Rabbit polyclonal anti-VP2 encoded by ALSV | This study | N/A |

*(Continued on next page)*

| ***Continued*** |  |  |
| --- | --- | --- |
| REAGENT or RESOURCE | SOURCE | IDENTIFIER |
| Bacterial and Virus Strains |  |  |
| ALSV | Wang et al., 2019 (1) | N/A |
| E. coli. (One Shot Stbl3 Chemically Competent) | Thermo Fisher Scientific | Cat# C737303 |
| E. coli. (Trans5α Chemically Competent Cell) | TransGen | cat# CD201-01 |
| Chemicals, Peptides, and Recombinant Proteins |  |  |
| Fetal bovine serum (FBS) | BBI | Cat# E600001 |
| Penicillin and streptomycin (PS) | Sangon | Cat# B540732 |
| Dulbecco’s modified Eagle’s medium (high glucose) | Sigma-Aldrich | Cat# R8758-500ML |
| Opti-MEM | Gibco | Cat# 31985-070 |
| Polyethylenimine Linear (PEI) MW40000 | YEASEN | Cat# 40816ES02 |
| Puromycin | YEASEN | Cat# 60210ES25 |
| Recombinant human IFN-β | PeproTech | Cat# 300-02BC |
| L-15 (Leibovitz) medium | Pricella | Cat# PM151010 |
| Tryptose phosphate broth | Solarbio | Cat# LA1660 |
| L-glutamine | Solarbio | Cat# G0200 |
| Fetal bovine serum | Sigma | Cat# F8318 |
| PBS | CELLCOOK | Cat# CM2018 |
| 0.25% Trypsin-EDTA | Procell | Cat# PB180226 |
| MG132 | Merck | Cat# 474787 |
| chloroquine (CQ) | MedChemExpress (MCE) | Cat# HY-17589A |
| 3-Methyladenine (3-MA) | Selleck | Cat# S2767 |
| Z-VAD-FMK | Selleck | Cat# S7023 |
| DMSO | Sigma-Aldrich | Cat# D8418 |
| FuniCut™ XbaI | YEASEN | Cat# 15033ES76 |
| Protease Inhibitor Cocktail | Selleck | Cat# B14002 |
| HaltTM Protease and Phosphatase Inhibitor Single-Use Cocktail | Thermo Fisher Scientific | Cat# 78442 |
| 6 × Protein Loading buffer | TransGen | cat# DL101-02 |
| BSA | VETEC | cat# V900933 |
| IMMOBILON WESTERN CHEMILUM HRP SUBSTRATE | Millipore | cat# WBKlS0100 |
| anti-Flag M2 Affinity Gel | Sigma-Aldrich | cat# A2220 |
| anti-HA Affinity Gel | Millipore | cat# E6779 |
| DAPI | YEASEN | Cat# 40728ES03 |
| poly-L-lysine | Sigma-Aldrich | Cat# P4832 |
| Paraformaldehyde (PFA) | Biotopped | cat# Top0382 |
| Triton X-100 | YEASEN | cat# 20107ES76 |
| MitoTracker® Red CMXRos | YEASEN | Cat# 40741ES50 |
| Critical Commercial Assays |  |  |
| Hieff Clone® Plus One Step Cloning Kit | YEASEN | Cat# 10911 |
| Pierce™ BCA Protein Assay Kits | Thermo Fisher Scientific | Cat# 23225 |
| EasyPure® RNA Kit | TransGen | cat# ER101 |

*(Continued on next page)*

| ***Continued*** |  |  |
| --- | --- | --- |
| REAGENT or RESOURCE | SOURCE | IDENTIFIER |
| Viral RNA extraction kit | TIANGEN | Cat# DP315 |
| cDNA Synthesis SuperMix | TransGen | cat# AT341 |
| Premix Ex Taq (Probe qPCR), Bulk | TaKaRa | cat# RR390L |
| Fast SYBR Green Master Mix | Roche | cat# 4913850001 |
| Dual-luciferase reporter assay | Promega | cat# E1910 |
| Nuclear and Cytoplasmic Protein Extraction Kit | Beyotime | cat# P0027 |
| Cell Mitochondria Isolation Kit | Beyotime | Cat# C3601 |
| Cell/Tissue Genomic DNA Extraction Kit | TIANGEN | Cat# DP304 |
| Experimental Models: Cell Lines |  |  |
| Human: HepG2 cells | ATCC | HB-8065 |
| Human: HEK293T cells | ATCC | CRL-3216 |
| Human: A549 cells | ATCC | CCL-185 |
| African green monkey (Chlorocebus sabaeus): Vero cells | JCRB | JCRB0111 |
| Human: STAT2^-/-^ cells | This study | N/A |
| IRE/CTVM19 cells | The Tick Cell Biobank | IRE/CTVM19 |
| Raw264.7 cells | ATCC | TIB-71 |
| Oligonucleotides |  |  |
| Forward primer for ALSV Segment 2 RT-qPCR:  GCTTGTGGTCATCATTATG | This paper | N/A |
| Reverse primer for ALSV Segment 2 RT-qPCR:  CTCTGCCACATACTGATG | This paper | N/A |
| 5’FAM/3’BHQ1-labeled probe primer for ALSV TaqMan qPCR:  CTCTCGTCAGCCATACCACCA | This paper | N/A |
| Forward primer for human IFIT1 RT-qPCR:  AAGGATAGTCTGGAGCAA | This paper | N/A |
| Reverse primer for human IFIT1 RT-qPCR:  CATAGGCTAGTAGGTTGTG | This paper | N/A |
| Forward primer for human IFIT2 RT-qPCR:  ATACATACCAAACAATGCCTAC | This paper | N/A |
| Reverse primer for human IFIT2 RT-qPCR:  GAGCCACAGCGTGTCCTA | This paper | N/A |
| Forward primer for human IFIT3 RT-qPCR:  ACACCAAACAATGGCTAC | This paper | N/A |
| Reverse primer for human IFIT3 RT-qPCR:  AGGATTCAGTCCCTTCTC | This paper | N/A |
| Forward primer for human IRAV RT-qPCR:  AGTAACGATCTGGATGCCCA | This paper | N/A |
| Reverse primer for human IRAV RT-qPCR:  ACATGCGTAGGTTGGCTTCT | This paper | N/A |
| Forward primer for human STAT1 RT-qPCR:  AAAGGAAGCACCAGAGCC | This paper | N/A |

*(Continued on next page)*

| ***Continued*** |  |  |
| --- | --- | --- |
| REAGENT or RESOURCE | SOURCE | IDENTIFIER |
| Reverse primer for human STAT1 RT-qPCR:  CCCACTATCCGAGACACC | This paper | N/A |
| Forward primer for human STAT2 RT-qPCR:  TCAGCCTGGACTTAGAGC | This paper | N/A |
| Reverse primer for human STAT2 RT-qPCR:  GTGATACAGGTCCTTGGTCT | This paper | N/A |
| Forward primer for human PGC1-α RT-qPCR:  CAGTCGCAGTCACAACAC | This paper | N/A |
| Reverse primer for human PGC1-α RT-qPCR:  GGCTTTATGAGGAGGAGT | This paper | N/A |
| Forward primer for human Nrf1 RT-qPCR:  GGAGTGACCCAAACCGAACA | This paper | N/A |
| Reverse primer for human Nrf1 RT-qPCR:  CGGGAGAAGAAGGCGAGT | This paper | N/A |
| Forward primer for human TFAM RT-qPCR:  CTTGGGAAGAACAAATGA | This paper | N/A |
| Reverse primer for human TFAM RT-qPCR:  ACACTCCTCAGCACCATA | This paper | N/A |
| Forward primer for human GAPDH RT-qPCR:  GAGTCAACGGATTTGGTCGT | This paper | N/A |
| Reverse primer for human GAPDH RT-qPCR:  TGGGATTTCCATTGATGACA | This paper | N/A |
| Recombinant DNA |  |  |
| Plasmid: pWPI | Addgene | Cat# 12254 |
| Plasmid: pWPI-NSP1 | This paper | N/A |
| Plasmid: VR1012 | BioVector NTCC | Cat# VR1012 |
| Plasmid: Flag-NSP1 | This paper | N/A |
| Plasmid: Flag-NSP2 | This paper | N/A |
| Plasmid: Flag-VP1a | This paper | N/A |
| Plasmid: Flag-VP1b | This paper | N/A |
| Plasmid: Flag-VP2 | This paper | N/A |
| Plasmid: Flag-VP3 | This paper | N/A |
| Plasmid: Flag-VP4 | This paper | N/A |
| Plasmid: pCDNA3.1-HA-STAT1-Myc | MIAOLING BIOLOGY | Cat# P4263 |
| Plasmid: pCDNA3.1-HA-STAT2-Myc | MIAOLING BIOLOGY | Cat# P4105 |
| Plasmid: pCDNA3.1-HA-STAT2-F175A/R176A-Myc | This paper | N/A |
| Plasmid: HA-mSTAT2-VR1012 | This paper | N/A |
| Plasmid: ISRE-luc | BioVector NTCC | Cat# pGL4.45[luc2P/ISRE/Hygro] |
| Plasmid: pGL4.74 [hRluc/TK] | BioVector NTCC | Cat# 40856081441 |
| Plasmid: pMD2.G | Addgene | Cat# 12259 |
| Plasmid: psPAX2 | Addgene | Cat# 12260 |

*(Continued on next page)*

| ***Continued*** |  |  |
| --- | --- | --- |
| REAGENT or RESOURCE | SOURCE | IDENTIFIER |
| Plasmid: LentiCRISPRv2 | Addgene | Cat# 52961 |
| Plasmid: pLenti-STAT2-sgRNA | Beyotime | Cat# L19990 |
| Plasmid: pSTAT1-Luc | BioVector NTCC | Cat# 8642661 |
| Plasmid: pSTAT2-Luc |  | N/A |
| Plasmid: GFP-LC3B | BioVector NTCC | Cat# BioVector1058 26-9 |
| Software |  |  |
| FlowJo software | BD Biosciences | Ver. 10 https://www.flflowjo.com/  solutions/flflowjo |
| ImageJ | Schneider et al., 2012 (2) | https://imagej.nih.gov/ij/ |
| Prism 9.0.2 | GraphPad Software | https://www.graphpad.com/scientific software/prism/ |
| Other |  |  |
| 0.45-mm pore size filter | Merck | Cat# SLGVR33RB |
| PVDF membranes | Millipore | cat# IPVH00010 |
| Nunc™ Cell culture tube | Thermo Fisher Scientific | Cat# 156758 |
| LSRFortessa | BD Biosciences | N/A |
| StepOne Plus Real-Time PCR system | Applied Biosystem | N/A |
| Confocal Microscopy | OLYMPUS | FV3000 |

1. Wang ZD, Wang B, Wei F, Han SZ, Zhang L, Yang ZT, Yan Y, Lv XL, Li L, Wang SC, Song MX, Zhang HJ, Huang SJ, Chen J, Huang FQ, Li S, Liu HH, Hong J, Jin YL, Wang W, Zhou JY, Liu Q. 2019. A New Segmented Virus Associated with Human Febrile Illness in China. N Engl J Med 380:2116-2125. <http://dx.doi.org/10.1056/NEJMoa1805068>.

2. Schneider CA, Rasband WS, Eliceiri KW. 2012. NIH Image to ImageJ: 25 years of image analysis. Nat Methods 9:671-5. <http://dx.doi.org/10.1038/nmeth.2089>.
